# Structural evolution in thermoelectric zinc antimonide thin films studied by *in situ* X-ray scattering techniques

**DOI:** 10.1107/S2052252521002852

**Published:** 2021-04-13

**Authors:** Lirong Song, Martin Roelsgaard, Anders B. Blichfeld, Ann-Christin Dippel, Kirsten Marie Ørnsbjerg Jensen, Jiawei Zhang, Bo B. Iversen

**Affiliations:** a Center for Materials Crystallography, Department of Chemistry and iNANO, Aarhus University, Aarhus C, DK-8000, Denmark; b Deutsches Elektronen-Synchrotron DESY, D-22607 Hamburg, Germany; c Department of Chemistry, University of Copenhagen, Copenhagen Ø, DK-2100, Denmark

**Keywords:** zinc antimonide thin films, structural evolution, *in situ* X-ray diffraction, *in situ* X-ray total scattering

## Abstract

Zinc antimonides have been widely studied owing to their outstanding thermoelectric properties. Unlike in the bulk state, where various structurally unknown phases have been identified through their specific physical properties, a number of intermediate phases in the thin-film state remain largely unexplored. Here, *in situ* X-ray diffraction and X-ray total scattering are combined with *in situ* measurement of electrical resistivity to monitor the crystallization process of as-deposited amorphous Zn-Sb films during post-deposition annealing. The as-deposited Zn-Sb films undergo a structural evolution from an amorphous phase to an intermediate crystalline phase and finally the ZnSb phase during heat treatment up to 573 K. An intermediate phase (phase B) is identified to be a modified β-Zn_8_Sb_7_ phase by refinement of the X-ray diffraction data. Within a certain range of Sb content (∼42–55 at%) in the films, phase B is accompanied by an emerging Sb impurity phase. Lower Sb content leads to smaller amounts of Sb impurity and the formation of phase B at lower temperatures, and phase B is stable at room temperature if the annealing temperature is controlled. Pair distribution function analysis of the amorphous phase shows local ordered units of distorted ZnSb_4_ tetrahedra, and annealing leads to long-range ordering of these units to form the intermediate phase. A higher formation energy is required when the intermediate phase evolves into the ZnSb phase with a significantly more regular arrangement of ZnSb_4_ tetrahedra.

## Abstract 2

Phase evolution was monitored during annealing of as-deposited amorphous zinc antimonide thin films using *in situ* X-ray diffraction and *in situ* X-ray total scattering coupled with electrical resistivity measurements.

## 1. Introduction   

Previous structural studies of thin films have mostly focused on the final thermodynamically stable phases in the crystallized films after annealing. However, to obtain growth control and understand the structural chemistry, it is also important to understand the metastable intermediate phases during the crystallization process (Bauers *et al.*, 2015 ▸; Kurzman *et al.*, 2015 ▸; Shoemaker *et al.*, 2014 ▸) as well as acquire information on the critical temperature at which the amorphous films crystallize or the crystalline phase transition temperatures in the crystallized films. For structural analysis, X-ray diffraction (XRD) provides quantitative structural information on the crystalline phases, whereas pair distribution function (PDF) provides information about the local structure at the atomic scale for both amorphous and crystalline phases (Billinge & Levin, 2007 ▸; Tyrsted *et al.*, 2012 ▸; Jensen *et al.*, 2015 ▸; Bøjesen *et al.*, 2016 ▸; Bøjesen & Iversen, 2016 ▸). The PDF technique was initially introduced to study thin films in normal incidence (NI) providing straightforward data modeling (Jensen *et al.*, 2015 ▸). Later, grazing incidence thin-film PDF (GI-tfPDF) was successfully developed providing a much stronger signal-to-noise ratio by reducing the penetration of the incident beam into the substrate while also increasing surface sensitivity and signal intensity, but at the expense of more complex experimental conditions (Dippel *et al.*, 2019 ▸; Roelsgaard *et al.*, 2019 ▸) such as much higher requirements for the alignment procedures with high-energy photons required to obtain *Q* values in the 20–30 Å^−1^ range. In this work, a combination of *in situ* X-ray diffraction, *ex situ* and *in situ* GI-PDF analyses was performed to monitor the phase evolution in as-deposited amorphous Zn-Sb thin films. Here we introduce *in situ* thin-film PDF data collection and demonstrate the possibility of obtaining quantitative data suitable for structural refinements. In addition, measurement of resistivity *in situ* is a powerful tool to observe intermediate phases. This technique has been applied to reveal phase changes in Cr_2_AlC thin films without the measurement of PDF data (Stelzer *et al.*, 2019 ▸). Here we demonstrate simultaneous *in situ* measurement of electrical resistivity and NI-PDF to observe the structural changes in Zn-Sb thin films.

The Zn-Sb material system studied here has attracted steadily increasing research interest since the discovery of a high thermoelectric (TE) figure of merit *zT* of 1.3 at 673 K in β-Zn_4_Sb_3_ by Caillat *et al.* (1997 ▸). Zinc antimonide materials also play a role in many other fields of application such as lithium- and sodium-ion batteries and phase change memory materials (Zhao & Cao, 2001 ▸; Nie *et al.*, 2016 ▸; Park *et al.*, 2007 ▸). Aside from the interest in the excellent thermoelectric properties, the extreme structural diversity of the zinc-antimony binary phase diagram has also been intensively investigated. Recently, Lo *et al.* (2018 ▸) updated the Zn–Sb phase diagram, and White *et al.* (2018 ▸) explored the Li–Zn–Sb phase diagram using a solution-phase method. It is now established that there exists a large number of unique Zn–Sb phases within a narrow compositional range (50–60 at% Zn) including ZnSb (Telkes, 1947 ▸), Zn_8_Sb_7_ (Pomrehn *et al.*, 2011 ▸; Wang & Kovnir, 2015 ▸), Zn_9_Sb_7_ (He *et al.*, 2015 ▸), Zn_4_Sb_3_ (Caillat *et al.*, 1997 ▸) and Zn_3_Sb_2_ (Lo *et al.*, 2017 ▸; Boström & Lidin, 2004 ▸). Among these, ZnSb and β-Zn_4_Sb_3_ are the two well known stable bulk phases at room temperature (RT, ∼300 K), both exhibiting excellent TE properties (Telkes, 1947 ▸; Caillat *et al.*, 1997 ▸). β-Zn_8_Sb_7_ is a stoichiometric compound (line phase) with an orthorhombic crystal structure (space group *Pmn*2_1_) with the lattice parameters *a* = 15.029 (1), *b* = 7.7310 (5), and *c* = 12.7431 (9) Å (Wang & Kovnir, 2015 ▸). β-Zn_8_Sb_7_ was obtained in the form of single crystals using the flux method or polycrystalline bulk samples through solid-state reaction followed by quenching. The electrical resistivity and Seebeck coefficient of β-Zn_8_Sb_7_ at 400 K are 15 mΩ cm and 275 µV K^−1^, respectively, which are comparable to the values of ZnSb (6.6 mΩ cm and 284 µV K^−1^), and its low total thermal conductivity (0.60 W m^−1^ K^−1^) is similar to β-Zn_4_Sb_3_ (0.75 W m^−1^ K^−1^), leading to a *zT* value of 0.33 at 400 K (Wang & Kovnir, 2015 ▸), which is comparable to ZnSb (*zT* = 0.37 at 400 K) (Xiong *et al.*, 2013 ▸) and Zn_4_Sb_3_ (*zT* = 0.40 at 400 K) (Toberer *et al.*, 2010 ▸). In the case of thin films, Sun *et al.* (2012 ▸) observed two unknown metastable phases in Zn-Sb films deposited at temperatures above RT. Chen *et al.* (2014 ▸) and Saito *et al.* (2015 ▸) investigated the crystallization behaviors of Zn*_x_*Sb_100−*x*_ films via characterization of the annealed films with X-ray diffraction (XRD) at RT, and it was claimed that the ZnSb metastable phase could be indexed as JCPDS No. 40–809 (orthorhombic crystal structure with space group *P*, lattice parameters: *a* = 10.32, *b* = 15.37, *c* = 7.49 Å) in the XRD database (Zielinski & Calka, 1982 ▸), whereas its detailed atomic positions, site occupancies and atomic displacement parameters (ADPs) remain unknown. So far no clear crystal structure refinement of this metastable phase has been reported. In this article we investigate the structural phase evolution during thermal annealing for as-deposited Ag-doped and undoped zinc antimonide films, since Ag doping has been demonstrated as an effective way to improve the TE performance as well as the thermal stability (Xiong *et al.*, 2013 ▸; Song *et al.*, 2017 ▸, 2018 ▸, 2019 ▸).

## 2. Experimental   

### 2.1. Sample preparation   

#### 2.1.1. Synthesis of bulk sputtering targets   

The sputtering targets were synthesized by the spark plasma sintering (SPS) method previously reported (Yin *et al.*, 2014 ▸; Blichfeld & Iversen, 2015 ▸; Song *et al.*, 2018 ▸, 2020 ▸). To make the undoped targets (Ud1, Ud2 and Ud3), Zn powders (purity 99.99%, Merck KGaA) and Sb powders (purity 99.5%, Sigma–Aldrich Chemie GmbH) were weighed according to the stoichiometric ratio of the corresponding nominal composition shown in Table S1 of the supporting information. For the Ag-doped targets (Ad1, Ad2 and Ad3), Ag powders (purity 99.99%, Sigma–Aldrich Chemie GmbH) were also added corresponding to the Ag-doping content. A mixer (SpectroMill, Chemplex Industries, Inc.) was used to blend the powders for 15 min. Then the powders were loaded into a graphite die with a 25.4 mm inner diameter. To avoid Zn migration induced by the large electric current during sintering, both the top and the bottom sides of the graphite punches were electrically insulated by a thin layer of boron nitride (BN) spray. An SPS-515 instrument (SPS Syntex Inc. Japan) was employed to compact and sinter the powders under vacuum. The applied pressure and temperature programs are given in Table S1. All the samples were cooled naturally while the pressure was slowly released.

#### 2.1.2. Growth of thin films   

The thin films were grown by single-target direct current (DC) magnetron sputtering on fused silica substrates. The substrates were cleaned by ultra-sonication in acetone and then in ethanol for around 15 min each. The base pressure of the sputtering chamber was about 10^−7^ mbar before releasing the sputtering gas argon (Ar, high-purity 99.9999%) into the chamber. The glow plasma discharge was created using the DC power supply. Prior to the real sputtering for thin-film deposition, a pre-sputtering process with a shutter shielding the substrate was performed to remove the contaminants on the target surface and make the plasma stable. Afterwards, for thin-film deposition with the shutter opened, the sputtering power, Ar flow rate and deposition pressure were set to 10 W, 8 standard cubic centimetres per minute (sccm) and 0.6 Pa, respectively. More specific information for all the thin-film samples can be found in Tables 1 ▸ and S2.

### 2.2. Characterization of Zn-Sb thin films   

#### 2.2.1. X-ray diffraction measurements   

The *in situ* XRD measurements for the as-deposited thin-film samples were carried out on a domed heating stage (Anton Paar DHS 1100) using a Rigaku SmartLab diffractometer with parallel-beam (PB) optics. The XRD patterns were collected in 2θ scan mode with a fixed incidence angle at 10°. After finishing each scan, approximately 1 min is needed before starting a new scan. Those reported are the temperatures at the beginning of a scan. Other experimental conditions of the *in situ* XRD measurements for each thin-film sample are summarized in Tables 2 ▸ and S3.

#### 2.2.2. X-ray total scattering measurements   

X-ray total scattering data were collected at the P07-EH2 endstation at PETRA III, DESY, Hamburg, Germany. For three Ag-doped thin-film samples (ZSA1_423K, ZSA2 and ZSA3), PDF measurements were conducted with an X-ray wavelength of 0.1260 Å in grazing incidence (GI) geometry with an incidence angle of 0.020° and with a 2 × 30 µm (FWHM, full width at half-maximum) focused X-ray beam, forming a footprint of *l* = 2 × 10^−3^ mm/sin (0.02°) ≃ 5.7 mm. The exposure time was 1 s. Detailed description of the grazing-incidence thin-film PDF setup is given elsewhere (Dippel *et al.*, 2019 ▸; Roelsgaard *et al.*, 2019 ▸). The detector was placed 397.49 mm downstream of the sample, with the X-ray beam centered on the detector, while masking out the bottom half of the detector covered by the horizontal shadow of the substrate for the GI measurements. To account for thermal expansion on heating, a second experiment was performed using the reflection of the direct beam to continuously follow the displacement to form an educated guess, which is on the order of 100 µm over the temperature range. This is much larger than the tolerance of approximately 1.5 µm in the vertical direction before the X-ray spills over the length of the substrate at 1 × 10 × 10 mm (*h* × *l* × *d*). For the PDF refinements the instrument resolution was accounted for by refinement on a CeO_2_ powder standard spread on an identical substrate, and in a Kapton capillary for the normal incidence (NI) experiments. For the *ex situ* GI-PDF measurements on the ZSA1_423K thin film, the hotplate was pre-heated to the target temperature, and then the thin-film sample was placed on the hotplate for a few minutes, taken off and the data were measured at the beamline. The NI thin-film PDF setup (Jensen *et al.*, 2015 ▸) was used to perform the *in situ* experiments with a heating ramp of 1 K min^−1^ for the two undoped thin-film samples (ZS2 and ZS3_373K). An exposure time of 60 s, X-ray wavelength of 0.155975 Å and a sample-to-detector distance of 315.492 mm were used. In addition to the PDF measurements, four-point resistivity measurements were carried out simultaneously using this setup. More details of the setup are provided in Figs. S1 and S2 of the supporting information. Table 3 ▸ provides information about the PDF measurements for the three Ag-doped and two undoped thin-film samples.

The two-dimensional diffraction patterns obtained were integrated in *pyFAI* (Ashiotis *et al.*, 2015 ▸), transformed into PDFs in *xPDFSuite* (Yang *et al.*, 2015 ▸) using *PDFGetX3* (Juhás *et al.*, 2013 ▸) and modeled in *PDFGui* (Farrow *et al.*, 2007 ▸). The *Q*_max_ value was 17 Å^−1^ for the NI-PDFs obtained and 21 Å^−1^ for the GI-PDFs. In order to acquire the background originating from the fused silica substrate and subtract it from the total scattering data, an identical clean fused silica substrate was aligned in the beam and data were measured at temperatures close to the individual frames, *i.e.* for the GI measurements the scattering pattern from the substrate was measured at RT, 473 and 573 K. *PDFGetX3* uses the latest data reduction methods and is well suited for the case of separating very weak signals from the total signals (Terban *et al.*, 2015 ▸). Wth regards to modeling of the PDF data in *PDFgui*, the scale factor, lattice parameters, symmetry-allowed atomic positions, atomic displacement parameters (ADPs) and correlated motion (*delta*2 parameter) were refined. The particle diameter (spdiameter) was also refined if the resulting value was reasonable.

#### 2.2.3. Electron microscopy analysis   

An FEI Nova NanoSEM 600 Scanning Electron Microscope (SEM) equipped with an energy-dispersive X-ray spectroscopy (EDX) detector was used to determine the elemental composition as well as to measure the film thickness for the thin-film samples (see Tables 1 ▸ and S2). For compositional analysis, three regions were detected for each sample and the average of the three results is given in Table 1 ▸. Generally, a higher Zn content in the sputtering target leads to more Zn in the thin film. SEM-EDX results reveal the presence of Ag in the Ag-doped film samples. Due to the low Ag content level, the estimated Ag compositions are not very accurate. The surface morphologies of two representative thin films were observed by SEM (see Fig. S3).

## 3. Results and discussion   

### 3.1. Phase evolution monitored via *in situ* X-ray diffraction during annealing   

**Figure 1:**
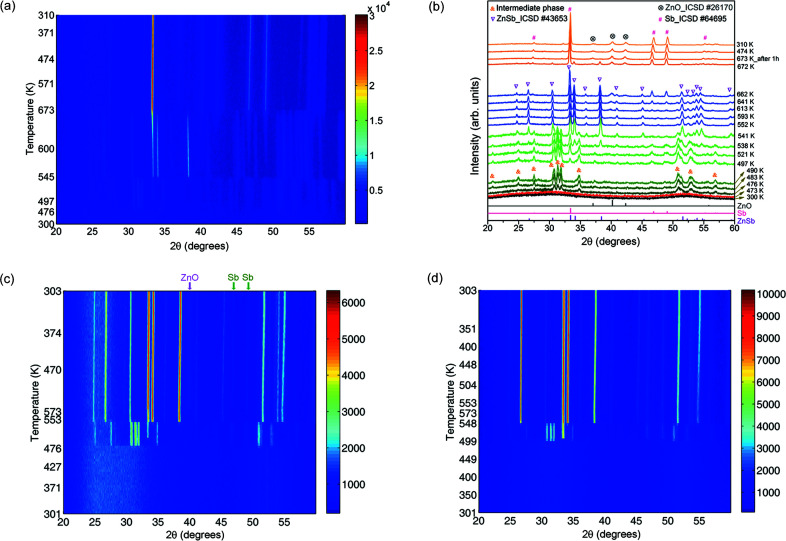
In-house *in situ* XRD data (Co *K*α source): (*a*) contour plot of temperature resolved data and (*b*) diffractograms at selected temperatures for the ZSA1_373K thin film; contour plots for the (*c*) ZSA1_RT and (*d*) ZSA2 thin films.

We performed in-house *in situ* XRD measurements on one as-deposited Ag-doped thin-film sample of ZSA1_373K grown at 373 K by sputtering the (Zn_0.99_Ag_0.01_)_4_Sb_3_ target (Ad1). *In situ* XRD patterns were collected as a function of temperature, from RT to 673 K in air. Figs. 1 ▸(*a*)–1(*b*) show that the as-grown thin film is still amorphous, and it starts to crystallize in a metastable phase as the temperature is increased to around 476 K. Fig. 1 ▸(*b*) clearly shows that the peak intensities of the metastable phase increase with increasing temperature in the initial stage of transition from the amorphous phase to the metastable phase, indicating that there is less amorphous constituent present at higher temperatures. When the thin film is heated to ∼497 K, the ZnSb phase emerges, which gradually becomes the main phase. After annealing at 673 K for 1 h the thin film contains Sb as the main phase as well as a trace amount of ZnO, resulting from the decomposition of the ZnSb phase in air. We therefore conducted *in situ* XRD measurements up to 573 K in air for the other three Ag-doped thin-film samples of ZSA1_RT, ZSA1_423K and ZSA1_473K grown at different substrate temperatures by sputtering the same target of (Zn_0.99_Ag_0.01_)_4_Sb_3_ (Ad1), as well as for two thin-film samples of ZSA2 and ZSA3 grown using the sputtering targets Zn_0.99_Ag_0.01_Sb (Ad2) and Zn_0.98_Ag_0.02_Sb (Ad3), respectively. As shown in Figs. 1 ▸(*c*)–1(*d*) and Figs. S4–S5, all five of these Ag-doped thin films undergo the same phase evolution from the amorphous phase to the metastable phase and eventually to ZnSb and Sb. The four thin films made by sputtering the (Zn_0.99_Ag_0.01_)_4_Sb_3_ target have a starting composition of (Zn+Ag):Sb = 58:42, whereas the starting composition ratio (Zn+Ag):Sb for the two thin films deposited using the Zn_1−*x*_Ag*_x_*Sb targets (*x* = 0.01 and 0.02) is 45:55. The as-deposited thin films with a starting Sb composition of 42–55 at% show the same trend of phase evolution, *i.e.* amorphous → metastable → ZnSb.

In addition, the starting film composition has an effect on the temperatures at which the individual phases form and disappear. In Table 4 ▸, we compare the five Ag-doped films having the same temperature profile used for the *in situ* XRD measurements in air. The three Ag-doped thin films made by sputtering the (Zn_0.99_Ag_0.01_)_4_Sb_3_ target show the metastable phase appearing at around 480 K, which is lower than ∼500 K for the two thin films (ZSA2 and ZSA3) deposited using the Zn_1−*x*_Ag*_x_*Sb (*x* = 0.01 and 0.02) target. This indicates that lower Sb content in the sputtering target or as-deposited film leads to a lower temperature for the formation of the metastable phase. However, the Sb content has no distinct influence on the temperature of formation of the ZnSb phase (*i.e.* ∼540 K). For the three Ag-doped thin films made by sputtering the (Zn_0.99_Ag_0.01_)_4_Sb_3_ target, the Sb phase disappears within a short time after the ZnSb phase forms and gradually reappears at the end [Figs. 1 ▸(*c*) and S4], whereas the Sb phase remains present from the formation of the metastable phase in the two thin films of ZSA2 and ZSA3 made by sputtering the Zn_1−*x*_Ag*_x_*Sb (*x* = 0.01 and 0.02) target [Figs. 1 ▸(*d*) and S5]. The missing Sb diffraction peaks might be due to the reaction with excess amorphous Zn, and the reappearance takes place upon the gradual Zn oxidation at 573 K in air, which is indicated by the simultaneous appearance of the weak ZnO diffraction peak at about 40° and the two Sb peaks at ∼47 and 49° marked in Fig. 1 ▸(*c*).

**Figure 2:**
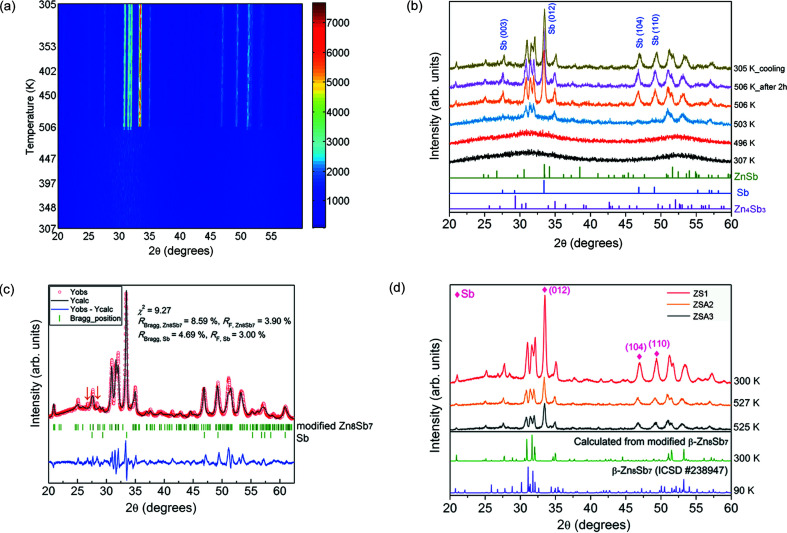
In-house *in situ* XRD data (Co *K*α source): (*a*) contour plot of temperature resolved data and (*b*) diffractograms at selected temperatures for the ZS1 thin film. (*c*) Calculated and observed diffraction patterns at 300 K of the annealed ZS1 thin film. The two-phase model does not fit the two weak peaks marked by arrows. (*d*) Experimental XRD patterns of the thin films (ZS1 at 300 K, ZSA2 at 527 K, ZSA3 at 525 K). The calculated XRD pattern of the modified β-Zn_8_Sb_7_ phase is shown in green. The purple curve shows the XRD pattern of the reported β-Zn_8_Sb_7_ phase (ICSD No. 238947).

In order to determine the crystal structure of the metastable phase, we attempted to obtain the metastable phase at RT so that better XRD data quality can be achieved using longer exposure time. Thus, one undoped thin-film sample of ZS1 grown using the ZnSb sputtering target (Ud1) was studied with in-house *in situ* XRD using a maximum temperature of only 506 K. After annealing at 506 K for 2 h and cooling to RT, the thin-film sample exhibits the metastable phase as well as Sb secondary phase [see Figs. 2 ▸(*a*)–2(*b*)]. The surface morphology of this annealed thin film is shown in Fig. S3(*a*). It is evident from Fig. 2 ▸(*c*) that the observed XRD pattern at 300 K can be well fitted by Rietveld refinement using the model with two phases of β-Zn_8_Sb_7_ (space group *Pmn*2_1_, ICSD No. 238947) and Sb (space group *R* 3 *m*, ICSD No. 64695). The refined parameters are shown in Table S4. The calcuated XRD pattern of the modified β-Zn_8_Sb_7_ phase obtained from the Rietveld refinement result is shown in green in Fig. 2 ▸(*d*). The experimental XRD patterns of the metastable phase in the thin films (ZS1, ZSA2 and ZSA3) match the calculated XRD pattern of the modified β-Zn_8_Sb_7_ phase better than the reported XRD pattern of β-Zn_8_Sb_7_. As shown in Fig. 2 ▸(*c*), the discrepancies in intensity for some peaks between the modified β-Zn_8_Sb_7_ phase and the metastable phase could be related to a certain degree of preferential growth in the two-dimensional thin film. The two weak peaks at ∼26.7 and 28.4° marked by the downward arrow symbol in Fig. 2 ▸(*c*) do not agree with the refinement model, possibly because of the presence of other unknown minor impurity phases.

In addition, the temperature-dependent electrical resistivity (ρ) and Seebeck coefficient (*S*) were measured for the annealed thin film ZS1, which shows the modified β-Zn_8_Sb_7_ phase and minor Sb phase. As shown in Fig. S6, the TE power factor (PF = *S* ^2^/ρ) is 0.5 µW cm^−1^ K^−2^ at 500 K, much lower than 9.5 µW cm^−1^ K^−2^ in the undoped ZnSb film (Song *et al.*, 2020 ▸) and 11 µW cm^−1^ K^−2^ in the Zn_4_Sb_3_ film (Sun *et al.*, 2012 ▸).

**Figure 3:**
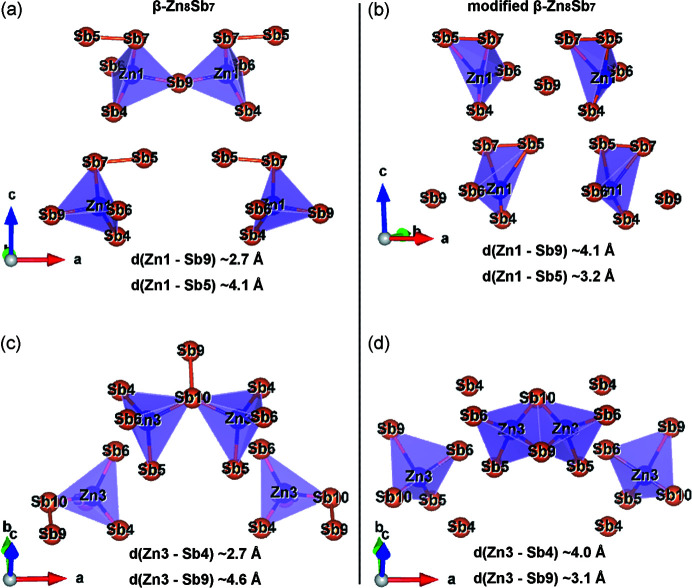
Zn1-centered tetrahedra for (*a*) the β-Zn_8_Sb_7_ phase and (*b*) the modified β-Zn_8_Sb_7_ phase. Zn3-centered tetrahedra for (*c*) the β-Zn_8_Sb_7_ phase and (*d*) the modified β-Zn_8_Sb_7_ phase.

The crystal structures of the reported β-Zn_8_Sb_7_ phase and the modified β-Zn_8_Sb_7_ phase obtained from our refinement on XRD data are shown in Fig. S7. Detailed information about atomic coordinates and selected interatomic distances for the two phases is given in Tables S5 and S6, respectively. In 2015, Wang & Kovnir (2015 ▸) reported the β-Zn_8_Sb_7_ phase with a non-centrosymmetric orthorhombic space group *Pmn*2_1_. The anionic framework consists of 28 Sb atoms, containing 10 Sb_2_^4−^ dimers and 8 isolated Sb^3−^ anions. There are 24 Zn atoms distributed over 6 fully occupied crystallographic sites (Zn1–Zn6) and the other 8 Zn atoms are distributed over 4 partially occupied sites (Zn7, Zn8, Zn9 and Zn10). A distinct difference between the β-Zn_8_Sb_7_ phase and the modified β-Zn_8_Sb_7_ phase is the change in the positions of the Zn1 and Zn3 atoms (see Table S5). Consequently, it can be seen in Fig. 3 ▸ that the interatomic distances of Zn1–Sb9 [∼4.1 Å, see Fig. 3 ▸(*b*)] and Zn3–Sb4 [∼4.0 Å, see Fig. 3 ▸(*d*)] in the modified β-Zn_8_Sb_7_ phase are significantly longer than those [∼2.7 Å, see Figs. 3 ▸(*a*) and 3(*c*)] in the β-Zn_8_Sb_7_ phase. This results in the Zn1-centered tetrahedron containing four vertices of Sb5, Sb4, Sb6 and Sb7 in the modified β-Zn_8_Sb_7_ phase [Fig. 3 ▸(*b*)] instead of Sb9, Sb4, Sb6 and Sb7 in the Zn_8_Sb_7_ phase [Fig. 3 ▸(*a*)], and the Zn3-centered tetrahedron has four vertices of Sb9, Sb5, Sb6 and Sb10 in the modified β-Zn_8_Sb_7_ phase [Fig. 3 ▸(*d*)] rather than those of Sb4, Sb5, Sb6 and Sb10 in the Zn_8_Sb_7_ phase [Fig. 3 ▸(*c*)].

### 3.2. Local structures revealed by PDF analysis   

*In situ* GI-PDF measurements were performed for the two Ag-doped thin films of ZSA2 and ZSA3 grown by sputtering the Zn_0.99_Ag_0.01_Sb and Zn_0.98_Ag_0.02_Sb targets, respectively. To assess the PDF in reciprocal space, plots of *F*(*Q*) and *S*(*Q*) were given to compare the experimental patterns with the theoretical patterns of the three phases of β-Zn_8_Sb_7_, ZnSb and Sb (see Figs. S8 and S9). The unwrapped 2D maps as well as the raw 2D diffraction patterns (Figs. S10 and S11) show no indication of preferred orientation at any stage.

**Figure 4:**
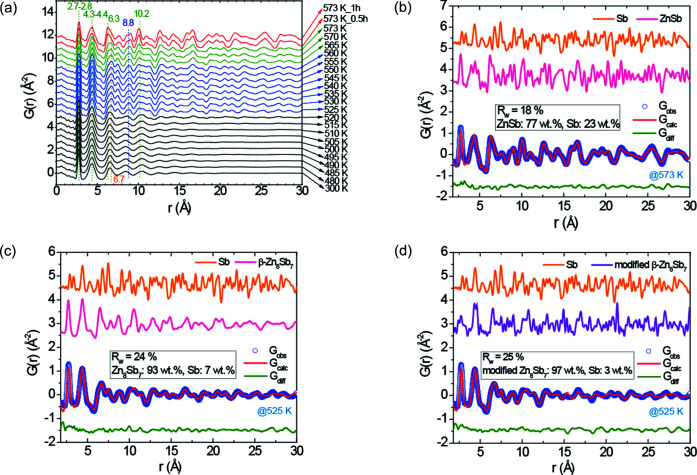
(*a*) *In situ* GI-PDFs from total scattering at some selected measurement temperatures for the ZSA2 film over the range 0–30 Å. The corresponding PDF data over 0–15 and 0–60 Å are shown in Fig. S13(*a*)–S13(*b*). Fits (red solid curve) to the experimental PDF data (blue open circles): (*b*) at 573 K after a dwell time of 1 h, and (*c*)–(*d*) at 525 K. In (*b*), (*c*) and (*d*), the difference between the experimental data and model fit is shown in green, and the refined parameters for the fits are given in Tables S7, S8 and S9.

Figs. 4 ▸(*a*) and S12(*a*) show that both ZSA2 and ZSA3 thin films exhibit similar structural evolution with increasing annealing temperature, which is consistent with the *in situ* XRD results shown in Figs. 1 ▸(*d*) and S5. The amorphous phase shows a local structure resembling that of the intermediate phase and has PDF peaks that terminate at significantly shorter distances compared with those of the two crystalline phases formed successively during annealing. The PDF data collected at 573 K after a dwell time of 1 h are modeled with a two-phase fit including the ZnSb and Sb phases. For these two thin films obtained by sputtering the Zn_1−*x*_Ag*_x_*Sb targets (*x* = 0.01, 0.02), the weight fractions of the Sb phase are estimated to be around 20 wt% [see Figs. 4 ▸(*b*) and S12(*b*)]. The small misfit in the first sharp peak may originate from amorphous ZnSb_4_ tetrahedral clusters that are not included in the models. For the PDF data obtained from either the ZSA2 thin film at 525 K or the ZSA3 thin film at 520 K, a two-phase fit with β-Zn_8_Sb_7_ and Sb was used [Figs. 4 ▸(*c*) and S12(*c*)]. The experimental PDF data look similar to the model fit, especially within the pair distance of 25 Å. Nevertheless, the agreement at larger distances is not as good as that below 25 Å. This suggests that the local crystal structure is similar, but there are some differences in the long-range structure. The reason for this might lie in the existence of different sizes of local domains with ordered structure in the thin film, which might be corroborated by the inhomogeneous grain size distribution observed by SEM in Fig. S3(*a*). As shown in Figs. 4 ▸(*c*), 4(*d*) and Fig. S14, the theoretical PDF patterns of the modified β-Zn_8_Sb_7_ phase and the β-Zn_8_Sb_7_ phase share similar features and generally many sharp peaks are located at similar positions, indicating the similarity in the strong atom pair correlations. This is consistent with the XRD data revealing the same space-group symmetry but slightly different structural parameters. Accordingly, the structure model with the modified β-Zn_8_Sb_7_ phase also provides a satisfactory fit to the observed PDF pattern for both ZSA2 and ZSA3 [see Figs. 4 ▸(*d*) and S12(*d*)].

Additional *ex situ* GI-PDF measurements were performed for the other half piece of the as-deposited thin-film sample ZSA1_423K obtained by sputtering the (Zn_0.99_Ag_0.01_)_4_Sb_3_ target. The resulting PDF patterns are shown in Fig. S15(*a*). The PDF data collected at 573 K were modeled with a two-phase fit including the ZnSb and Sb phases. As seen in Fig. S15(*b*), there is only ∼4 wt% Sb phase present after annealing at 573 K for 1 h, which is significantly less than ∼20 wt% Sb phase in the two annealed thin films by sputtering the Zn_1−*x*_Ag*_x_*Sb targets (*x* = 0.01, 0.02). As a result, the two shoulder peaks of the peak at 4.3–4.4 Å, which are observed in the PDF patterns at 573 K of the annealed ZSA1_423K thin film and originate from the ZnSb phase [see Fig. S15(*b*)], are not observed in the PDF patterns at 573 K of the two Ag-doped films (ZSA2 and ZSA3) by sputtering the Zn_1−*x*_Ag*_x_*Sb targets (*x* = 0.01, 0.02) [see Figs. 4 ▸(*a*), 4(*b*), S12(*a*) and S12(*b*)].

**Figure 5:**
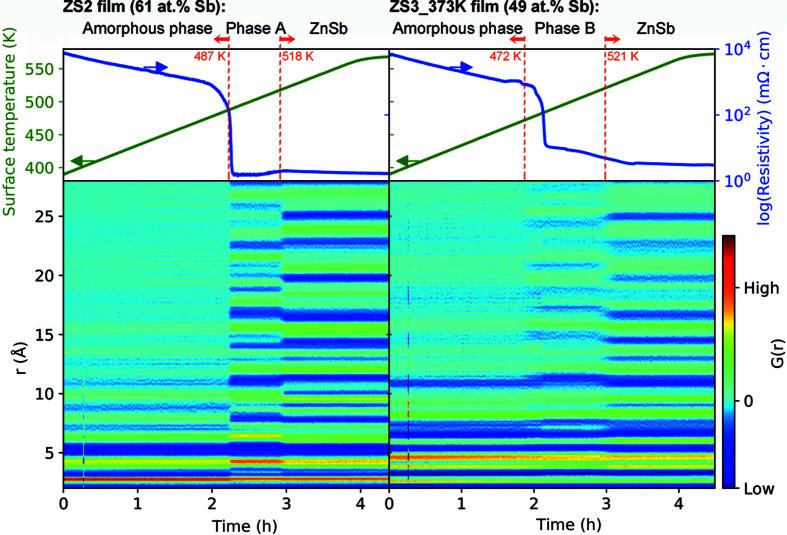
*In situ* thin-film NI-PDF and resistivity measured simultaneously for the as-deposited films: (left) ZS2 and (right) ZS3_373K. PDF patterns at selected surface temperatures are shown in Figs. S16 and S17.

For investigation of structural changes, it is beneficial if the electrical resistivity can be simultaneously monitored during the PDF measurements. We have successfully developed an approach to simultaneous *in situ* measurement of the resitivity and PDF data for thin films. This approach was applied to two undoped films of ZS2 obtained by sputtering the ZnSb target (Ud2) and ZS3_373K obtained by sputtering the Zn_4_Sb_3_ target (Ud3). The resulting data are plotted against the measurement time scale in Fig. 5 ▸. For both samples, structural transitions with time (*i.e.* temperature) are clearly observed, matching up perfectly with changes in the resistivity data. The steep drop in the resistivity corresponds with the transition from the amorphous to the metastable phases. In contrast with the ZS3_373K film with a starting Sb composition of 49 at%, the ZS2 film with a starting Sb composition of 61 at% exhibits obviously lower resistivity during the second stage of the intermediate phase formation, which is possibly due to larger amounts of the metallic phase of Sb formed (confirmed by the *in situ* XRD result shown in Figs. S18–S19) or larger grains induced by higher film thickness (Song *et al.*, 2020 ▸).

The PDF of the metastable phase (phase A) in the ZS2 film shows four peaks at around 2.8, 4.4, 6.8 and 12.8 Å, similar to that of the α-Zn_3_Sb_2_ phase [see Fig. S16(*a*)]. However, Fig. S19 shows that the XRD pattern of phase A is different from that of the α-Zn_3_Sb_2_ phase and also those of other known phases in the Zn-Sb binary system. Therefore, the crystal structure of phase A is yet to be determined and is beyond the reach of current techniques applied in this work. In addition, the experimental PDF data at 568 K of the ZS2 film are well described by including two phases (ZnSb and Sb) in the model (Fig. S20). The phase fractions of the ZnSb and Sb phases are 67 and 33 wt%, respectively.

As the ZS2 is much thicker than the ZS3_373K film, the PDF data obtained from ZS2 have a better signal-to-noise ratio than that of the data from the ZS3 film. Consequently, the final PDFs for the ZS3_373K film are of relatively poor quality as there is an Si—O peak at ∼1.6 Å [Fig. S17(*c*)], which can be attributed to the amorphous silica substrate (Biswas *et al.*, 2018 ▸). As seen in Fig. S16(*b*), the metastable phase (phase B) in the ZS3_373K film shows similar PDF features to those of the β-Zn_8_Sb_7_ phase. The peak at ∼4.4 Å for β-Zn_8_Sb_7_ is split into two peaks at 4.0 and 4.8 Å, possibly due to the absence of the 4.4 Å peak in amorphous silica (Biswas *et al.*, 2018 ▸). Moreover, the XRD pattern of phase B (Fig. S19) is the same as that of the modified β-Zn_8_Sb_7_ phase discussed above (Fig. 2 ▸). This undoped thin-film sample has a starting Sb composition of 49 at%. This further confirms the conclusions made based on the results of the Ag-doped Zn-Sb thin films discussed above: that the metastable phase in the Zn-Sb thin films with a starting Sb content of 42–55 at% is the modified β-Zn_8_Sb_7_ phase.

### 3.3. Discussion   

In addition to *in situ* XRD and thin-film GI-PDF, simultaneous *in situ* NI-PDF and electrical resistivity measurements have been carried out to investigate the crystallization process in Zn-Sb thin films. The approach of combining *in situ* total scattering PDF and electrical resistivity measurements shows great potential in tracking local structural changes during thermal treatment in correlation with changes in resistivity. This approach can be applied to many other material systems in which the different phases have differences in resistivity. The downside here is the use of transmission PDF, in which it is difficult to obtain a high-quality PDF. On the other hand, *in situ* GI-PDF resolves this issue but it is highly sensitive to small changes in experimental parameters, *e.g.* temperature. Although we achieved such measurement over a 300 K temperature range, the *in situ* GI-PDF measurements were without the simultaneous recording of resistivity data. Future technical advancements might enable a positional feedback loop to ensure higher reliability of the *in situ* GI-PDF data.

**Figure 6:**
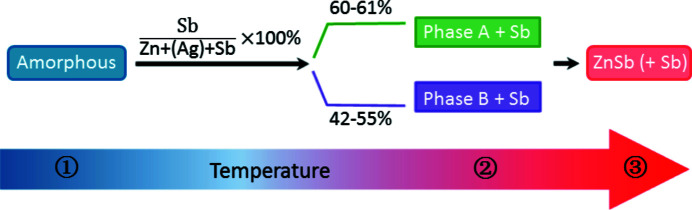
Proposed mechanism for the phase evolution during annealing of the as-deposited Zn-Sb thin films.

The as-deposited Zn-Sb thin films undergo a structural evolution from the amorphous phase to the intermediate phase (A or B) and finally the ZnSb phase. This indicates that less energy is required for the crystallization of phase A or B compared with the ZnSb phase. Here we propose a mechanism for the phase evolution, as shown in Fig. 6 ▸. When the starting Sb composition is 60–61 at%, the intermediate phase A starts to form together with the Sb phase. When the starting Sb content is lower (42–55 at.%), the intermediate phase B (Zn_8_Sb_7_: 46.7 at% Sb) and a small amount of the Sb phase emerge. As the temperature increases further (*T*_max_ = 573 K), then if the starting Sb content is as low as ∼42 at%, the Sb phase disappears possibly due to its reaction with excess amorphous Zn. The final phase will be only ZnSb if the post-deposition annealing (*T*_max_ = 573 K) is conducted under vacuum. Otherwise in air, oxidation takes place and the Sb phase reappears. The above analysis indicates that the starting film composition plays a key role in the evolution process during annealing of the amorphous Zn-Sb film.

**Figure 7:**
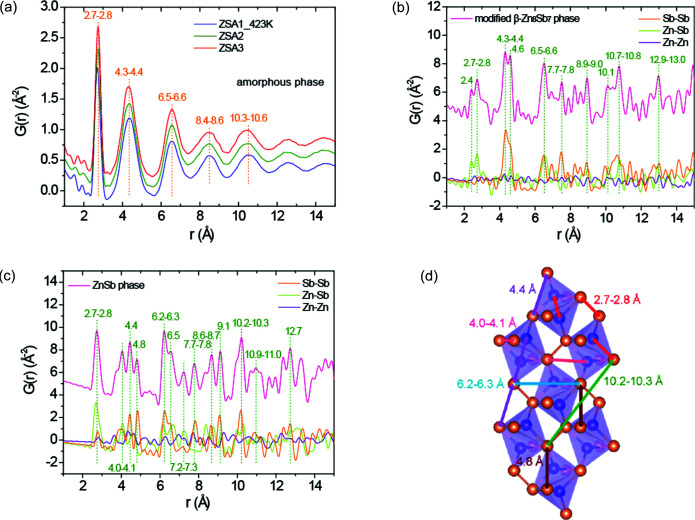
(*a*) Experimental GI-PDFs of the amorphous phase collected at 300 K for the three Ag-doped thin films. Total and partial theoretical PDFs of the (*b*) modified β-Zn_8_Sb_7_ and (*c*) ZnSb phases. (*d*) Selected pair distances corresponding to the peak positions indicated in (*c*).

The experimental GI-PDFs of the amorphous phase for the three Ag-doped thin films (ZSA1_423K, ZSA2 and ZSA3) are compared in Fig. 7 ▸(*a*). All the PDFs from the amorphous phase show two sharp PDF peaks at *ca* 2.8 and 4.4 Å, which are also present in the PDF from the intermediate phase and the final ZnSb phase. As indicated in Figs. 7 ▸(*b*)–7(*d*), these two peaks mainly correspond to the Zn–Sb distance and Sb–Sb edge distance in ZnSb_4_ tetrahedra, respectively. Therefore, we speculate that the as-deposited thin films contain ZnSb_4_ tetrahedra as the building units. Three broader medium-range peaks are observed before the structural coherence dies out above 11 Å, suggesting specific orientations of ZnSb_4_ tetrahedra that are favored over other orientations in the local clusters. Accordingly, the crystallization of the intermediate phase B may be driven by elongation of the range of ordered structures via a rearrangement of the building units. When phase B evolves towards the ZnSb phase, the isolated Sb^3−^ anions disappear and only Sb_2_^4−^ dimers remain. The ZnSb phase exhibits a much more regular structure with the ZnSb_4_ structural unit, which explains why a higher annealing temperature is needed to form this phase compared with phase B.

## 4. Conclusions   

The phase evolution during post-deposition annealing the Zn-Sb thin films was followed from the amorphous phase into the final product of the ZnSb phase via *in situ* XRD and *in situ* X-ray total scattering together with simulataneous resistivity measurements. Upon heating, an intermediate crystalline phase always forms before the ZnSb phase appears. Two distinct types of the intermediate phases (A or B) were observed for films with differing initial Sb content. When the Sb content stays within the 42–55 at% range, phase B is always observed. The intermediate phase coexists with a secondary Sb phase. In addition, phase B forms at lower temperatures for films with lower Sb content. Importantly, phase B can be stabilized at RT by controlling the annealing temperature, and is determined to be a modified β-Zn_8_Sb_7_ phase via Rietveld refinement of XRD data. Through PDF analysis, we speculate that the amorphous thin film shows local ordered clusters of ZnSb_4_ tetrahedra with specific favored orientations with respect to each other. The structural phase evolution is driven by obtaining an increasingly regular arrangement of distorted ZnSb_4_ tetrahedra. As such, the intermediate phase with lower formation energy occurs prior to the final ZnSb phase. In summary, the phase evolution of the amorphous thin film during post-deposition annealing can be effectively monitored using *in situ* XRD and PDF techniques in combination with measurement of electrical resistivity.

## Supplementary Material

Supporting information file. DOI: 10.1107/S2052252521002852/fc5044sup1.pdf

## Figures and Tables

**Table 1:** Sputtering target, substrate temperature *T*_sub_, film composition and measurements for all the thin-film samples NI and GI denote normal incidence and grazing incidence, respectively. The notations s1 and s2 mean two identical thin-film samples, and p1 and p2 mean two half pieces from one film sample.

Film sample	Target	*T*_sub_	Film composition (at%)			Measurement
			Zn	Sb	Ag	
ZSA1_RT	Ad1	RT	55.3 (6)	42.4 (5)	2.3 (1)	XRD
ZSA1_ 373K	Ad1	373 K	Near identical to ZSA1_RT			XRD
ZSA1_423K	Ad1	423 K	Near identical to ZSA1_RT			(p1) XRD + (p2) GI-PDF
ZSA1_473K	Ad1	473 K	Near identical to ZSA1_RT			XRD
ZSA2 (s1)	Ad2	RT	42.8 (4)	55.0 (5)	2.2 (1)	XRD
ZSA2 (s2)	Ad2	RT	43.2 (4)	54.5 (3)	2.3 (1)	GI-PDF
ZSA3 (s1)	Ad3	RT	42.8 (3)	54.6 (2)	2.5 (1)	XRD
ZSA3 (s2)	Ad3	RT	Near identical to ZSA3 (s1)			GI-PDF
ZS1	Ud1	RT	46.6 (4)	53.4 (4)	–	XRD
ZS2 (s1)	Ud2	RT	39.8 (5)	60.2 (5)	–	XRD
ZS2 (s2)	Ud2	RT	39.3 (3)	60.7 (3)	–	NI-PDF with resistivity
ZS3_373K	Ud3	373 K	51.5 (2)	48.5 (2)	–	NI-PDF with resistivity
ZS3_RT	Ud3	RT	50.9 (2)	49.1 (2)	–	XRD

**Table 2:** Experimental conditions of the *in situ* XRD measurements for the thin-film samples

Film sample	Temperature profile	Environment
ZSA1_373K	{\rm RT} \mathop \to \limits^{{\rm{10\,K\,min}}^{-1}} 473\,{\rm K}\mathop \to \limits^{{\rm{1\,K\,min}}^{-1}}673\,{\rm K}\mathop \to \limits^{{\rm{1\,h}}}673\,{\rm K}\mathop \to \limits^{{\rm{2\,K\,min}}^{-1}}310\,{\rm K}	Air

ZSA1_RT	{\rm RT} \mathop \to \limits^{{\rm{2\,K\,min}}^{-1}} 573\,{\rm K}\mathop \to \limits^{{\rm{2\,h}}}573\,{\rm K}\mathop \to \limits^{{\rm{2\,K\,min}}^{- 1}}{\rm RT}	Air
ZSA1_423K (p1)		
ZSA1_473K		
ZSA2 (s1)		
ZSA3 (s1)		

ZS1	{\rm RT}\mathop \to \limits^{{\rm{2\,K\,min}}^{- 1}}506\,{\rm K}\mathop \to \limits^{{\rm{2\,h}}}506\,{\rm K}\mathop \to \limits^{{\rm{2\,K\,min}}^{- 1}}{\rm RT}	Air

ZS2 (s1)	{\rm RT}\mathop \to \limits^{{\rm{1\,K\,min}}^{{\rm{ - 1}}}}573\,{\rm K}\mathop \to \limits^{{\rm{1\,h}}}573\,{\rm K}\mathop \to \limits^{{\rm{15\,K\,min}}^{- 1}}{\rm RT}	Dynamic vacuum

ZS3_RT	{\rm RT}\mathop \to \limits^{{\rm{20\,K\,min}}^{- 1}}473\,{\rm K}\mathop \to \limits^{{\rm{1\,K\,min}}^{- 1}}573\,{\rm K}\mathop \to \limits^{{\rm{1\,h}}}573\,{\rm K}\mathop \to \limits^{{\rm{15\,K\,min}}^{- 1}}{\rm RT}	Dynamic vacuum

**Table 3:** Details of the PDF measurements for thin-film samples

Film sample	Measurement	Temperature profile	Environment
ZSA1_423K (p2)	*Ex situ* GI-PDF	RT, 483 K, 553 K, 573 K_1 h, 573 K_2 h; heating rate: 2 K min^−1^	Air
	
ZSA2 (s2)	*In situ* GI-PDF	300\,{\rm K}\mathop \to \limits^{{\rm{5\,K\,min}}^{-1}}573\,{\rm K}\mathop \to \limits^{{\rm dwell}}573\,{\rm K}\mathop \to \limits^{{\rm{naturally\,cooling}}}{\rm RT}	Air
ZSA3 (s2)			
	
ZS2 (s2)	*In situ* NI-PDF with resistivity	423\,{\rm K}\mathop \to \limits^{{\rm 1\,K\,min}^{-1}}603\,{\rm K}\mathop \to \limits^{\rm naturally\,cooling}{\rm RT}	Air
ZS3_373K			

**Table 4:** Temperatures at which the metastable phase, the Sb phase or the ZnSb phase appear and disappear in the five Ag-doped thin films

Film sample		ZSA1_RT	ZSA1_423K	ZSA1_473K	ZSA2	ZSA3
Target		(Zn_0.99_Ag_0.01_)_4_Sb_3_			Zn_0.99_Ag_0.01_Sb	Zn_0.98_Ag_0.02_Sb
Film composition		(Zn+Ag):Sb = 58:42 = 1.38			(Zn+Ag):Sb = 45:55 = 0.82	
*T*_max_ (K)		573	573	573	573	573
	
Metastable phase						
*T*_appear_ (K)		480	478	484	499	497
*T*_disappear_ (K)		548	555	554	548	546
	
Sb						
*T*_appear_ (K)		497	499	498	499	497
*T*_disappear_ (K)		546	555	554	–	–
*T*_reappear_ (K)		573	573	573	–	–
	
ZnSb						
*T*_appear_ (K)		539	548	547	541	533
