# Supplementary material for: Structural evolution in thermoelectric zinc antimonide thin films studied by in situ X-ray scattering techniques
Source: IUCrJ. 2021 Apr 13;8(Pt 3):444–54. doi: 10.1107/S2052252521002852 (PMC8086166; doi:10.1107/S2052252521002852)
Supplement: Supplementary file 1 [file m-08-00444-sup1.pdf]

IUCrJ

Volume 8 (2021)

Supporting information for article:

Structural evolution in thermoelectric zinc antimonide thin films studied by *in situ* X-ray scattering techniques

Lirong Song, Martin Roelsgaard, Anders B. Blichfeld, Ann-Christin Dippel, Kirsten Marie Ørnsbjerg Jensen, Jiawei Zhang and Bo B. Iversen

Table S1 SPS parameters used for synthesizing the sputtering targets. For synthesizing the Ad#1 target, a Zn round foil (purity 99.95%), with a thickness of 0.15 mm and the same diameter as the inner diameter of the graphite die, was put on the bottom side between the powders and BN sprayed layer to compensate for the Zn loss during the synthesis.

Target	P (MPa)	t_{ramp} (min)	t_{sinter} (min)	T_{max} (K)	Composition	Note
Ad#1	100	10	5	673	$(Zn_{0.99}Ag_{0.01})_4Sb_3$	Zn foil at bottom
Ad#2	100	10	15	698	$Zn_{0.99}Ag_{0.01}Sb$	
Ad#3	100	10	15	698	$Zn_{0.98}Ag_{0.02}Sb$	
Ud#1	100	10	15	698	ZnSb	
Ud#2	50	10	5	673	ZnSb	
Ud#3	50	10	5	673	Zn_4Sb_3	No zinc foil

Table S2 The deposition time and film thickness for all the thin film samples.

Film sample	Deposition time (min)	Film thickness (nm)
**ZSA1_RT**	40	-
**ZSA1_ 373K**	60	-
**ZSA1 _423K**	54	-
**ZSA1 _473K**	60	-
**ZSA2 (s1)**	60	760
**ZSA2 (s2)**	60	760
**ZSA3 (s1)**	60	760
**ZSA3 (s2)**	60	760
**ZS1**	60	680
**ZS2 (s1)**	120	1325
**ZS2 (s2)**	120	1362
**ZS3_373K**	60	654
**ZS3_RT**	60	637

Table S3 Experimental parameters of the *in situ* XRD measurements for the thin film samples.

Film sample	Radiation wavelength λ (Å)	Scan range 2θ (°)	Scan speed (° min^{-1})
**ZSA1_373K**			
**ZSA1_ RT**			
**ZSA1_423K (p1)**			
**ZSA1_473K**	1.79 (Co Kα)	20-60	15
**ZSA2 (s1)**			
**ZSA3 (s1)**			
**ZS1**			
**ZS2 (s1)**	1.54 (Cu Kα)	20-50	10
**ZS3_RT**	1.54 (Cu Kα)	20-50	15

S1. Information of *in situ* normal incident (NI) thin-film PDF measurement setup (along with resistivity measurement)

The experiments of *in situ* thin-film PDF (tfPDF) in normal transmission mode were performed using a setup designed by Anders B. Blichfeld. As shown in Figure S1a-b, a large aluminum block was fitted with a resistive heat cartridge to ensure a large thermal mass and to minimize heat gradients through the sample. A slit was cut for the X-ray beam to pass through before hitting the sample. Stainless steel clamps covered in Kapton tape were used for supporting the film sample. In this work, the setup was also designed for a four-point resistivity measurement during *in situ* annealing (Figure S1c). The film surface was placed facing the detector and perpendicular to the incoming X-ray beam (Figure S1d). The thermocouple used for PID control was placed close to where the film was mounted inside the aluminum block. A second thermocouple was mounted on the film to monitor the surface temperature. The surface temperature has been calibrated with an IR-camera (Figure S1e). The calibration curve can be found in Figure S2.

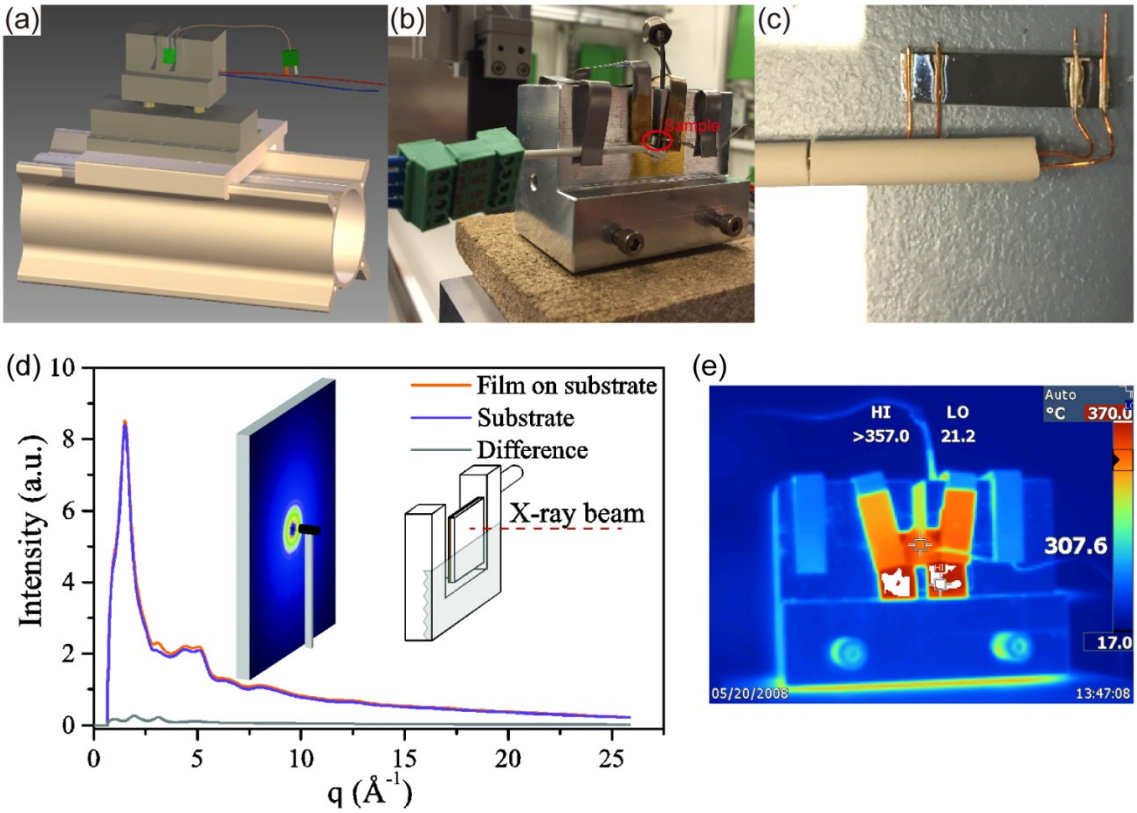

Figure S1 (a) The design drawings of the *in situ* NI-tfPDF setup. (b) Picture of the setup in use at the P07 beamline at the synchrotron PETRA III. (c) Picture of the sample with wires mounted for four-probe resistivity measurements. (d) shows the incoming X-ray beam and the detector. The scattering signals from a clean fused silica substrate and a thin film on the same fused silica substrate were collected. Then the PDF data of the film are the difference in the signals. For more information on the method of data collection using this setup, one can also refer to the previous report (Jensen *et al.*, 2015). (e) IR-camera reading of the surface temperature. The substrate mounted is coated with graphite spray, for resembling a black-body. The aluminum block is at the same temperature but appears to be around 20 °C because of the low emissivity.

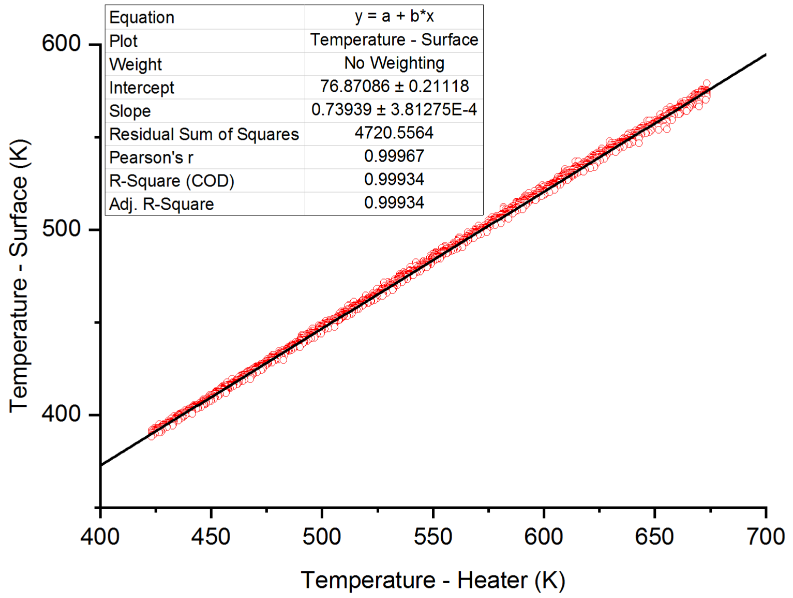

Figure S2 Temperature calibration curve for the surface temperature of a thin-film in the *in situ* NI-tfPDF setup. The surface temperature was measured by an IR-camera and correlated to the PID actual temperature reading.

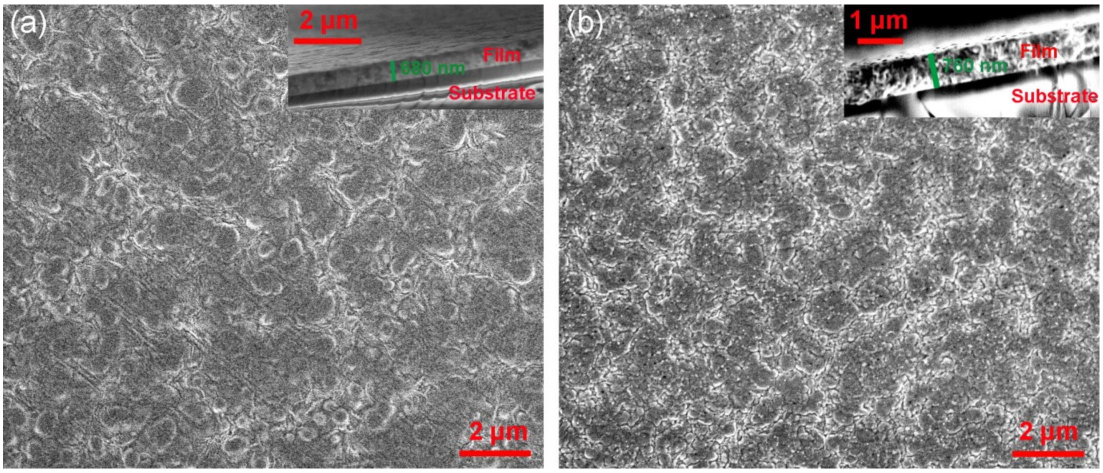

Figure S3 SEM surface images of the (a) ZS1 and (b) ZSA2 annealed thin film samples at 20000x magnification. The insets show the cross sections of the corresponding films.

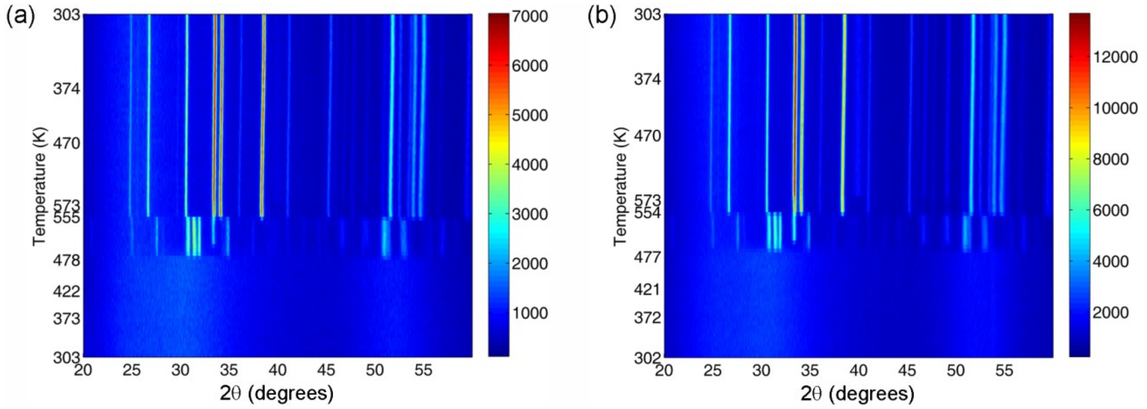

Figure S4 Contour plots of in-house *in situ* XRD data (Co-Kα source) for the as-deposited Ag-doped thin films: (a) ZSA1_423K, and (b) ZSA1_473K.

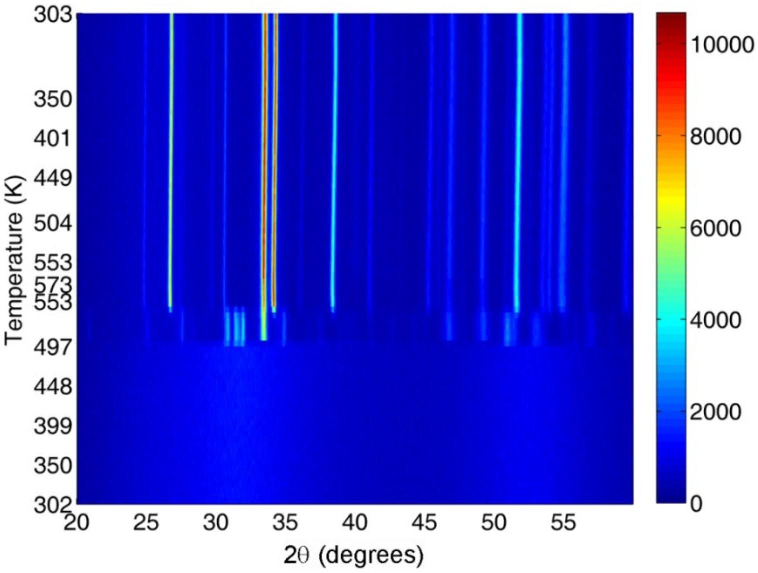

Figure S5 Contour plot of in-house *in situ* XRD data (Co-Kα source) for the as-deposited Ag-doped thin film ZSA3.

Table S4 Rietveld refinement details of the XRD data at 300 K for the annealed thin film ZS1. The XRD data were Rietveld refined using the FullProf program. The background was modelled using linear interpolation, and the Thompson-Cox-Hastings Pseudo-Voigt function convoluted with axial divergence asymmetry was used for modelling the peak-profile shape. The parameters refined include the scale factor, zero point, lattice constants, profile parameters, and atomic positions.

***T*(K)**	300
**$t_{exp.}$ (min)**	84
**No. of points**	8395
**No. of reflections (Zn_8Sb_7)**	580
**R_f (%) (Zn_8Sb_7)**	3.90
**R_{Bragg} (%) (Zn_8Sb_7)**	8.59
**R_p (%)**	21.1
**R_{wp} (%)**	23.2
**χ^2**	9.27
**Wt.% Zn_8Sb_7**	70.87(0.51)
**Wt.% Sb**	29.13(0.21)
***a* (Å), Zn_8Sb_7**	15.2080(10)
***b* (Å), Zn_8Sb_7**	7.4664(9)
***c* (Å), Zn_8Sb_7**	12.9629(13)
**Volume (Å^3), Zn_8Sb_7**	1471.9(2)

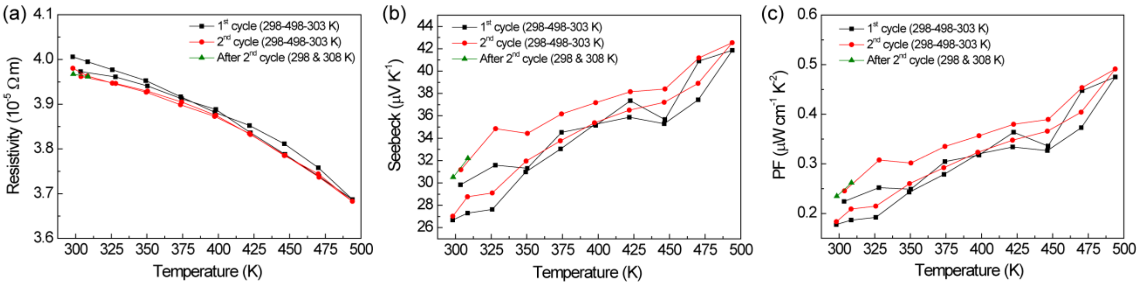

Figure S6 Temperature dependence of the (a) electrical resistivity, (b) Seebeck coefficient and (c) power factor for the annealed thin film sample ZS1. The temperature-dependent electrical resistivity and Seebeck coefficient were simultaneously measured on a ZEM-3 setup. The ZS1 film with the modified β-Zn_8Sb_7 phase exhibits slightly higher electrical resistivity and largely decreased Seebeck coefficient values in comparison with those of the undoped ZnSb film (Song *et al.*, 2020) and the Zn_4Sb_3 film (Sun *et al.*, 2012).

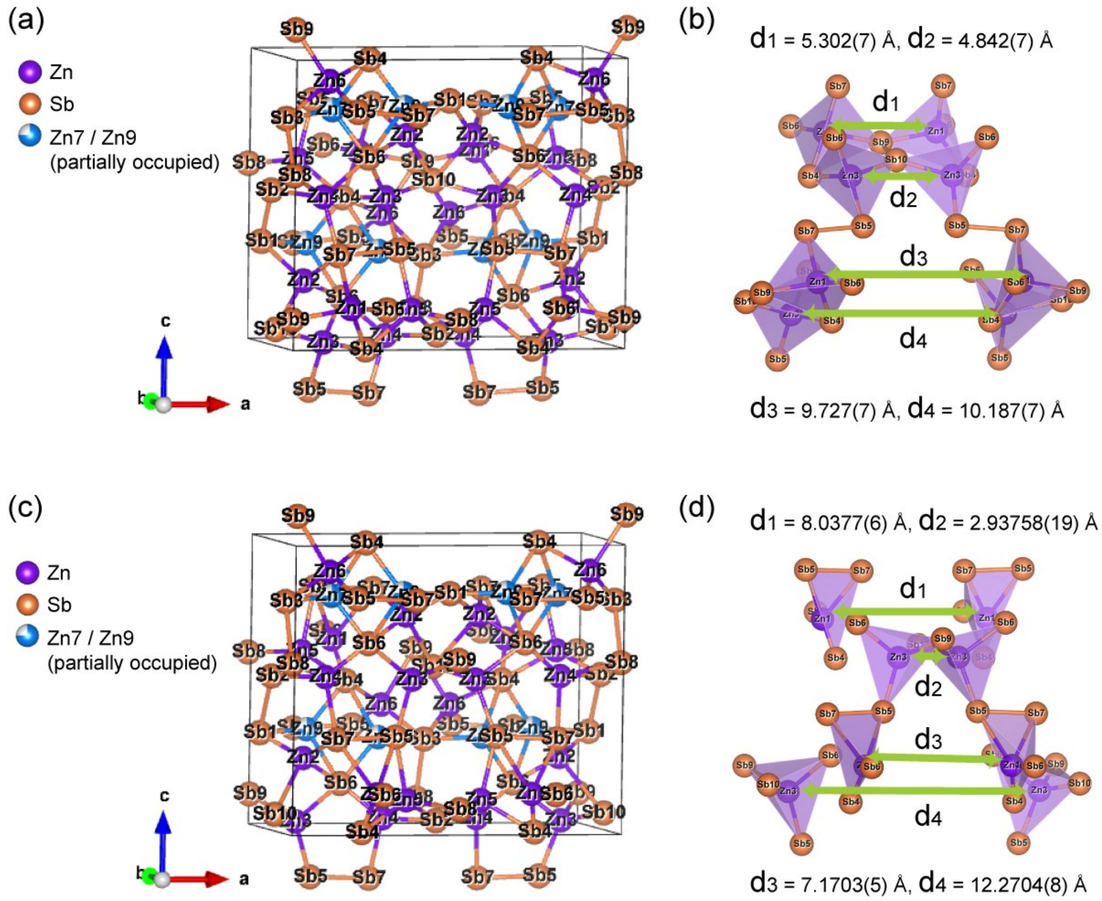

Figure S7 Crystalline phases of (a) β-Zn_8Sb_7 and (c) modified β-Zn_8Sb_7 in ball-and-stick representations. As the distances of the Zn7-Zn8 and Zn9-Zn10 sites are short (1.2-1.4 Å), only one of the two possible Zn atoms is present. Comparing with the Zn8 and Zn10 atoms in the structure, the Zn7 and Zn9 atoms have higher occupancies of 0.752(8) and 0.755(9), respectively. Hence here only the Zn7 and Zn9 atoms (in blue color) are shown in (a) and (c) for clarity. (b) and (d) display both Zn1-centered and Zn3-centered tetrahedra for the β-Zn_8Sb_7 and modified β-Zn_8Sb_7 phases, respectively. The notations of d_1, d_2, d_3 and d_4 indicated in both (b) and (d) are the corresponding Zn-Zn interatomic distances.

Table S5 Comparison of atomic coordinates between the β-Zn_8Sb_7 phase (*a* = 15.0291(10), *b* = 7.7310(5), *c* = 12.7431(9)) and the modified β-Zn_8Sb_7 phase (*a* = 15.2080(10), *b* = 7.4664(9), *c* = 12.9629(13)).

Atom	*x*		*y*		*z*	
	**β-Zn_8Sb_7**	**modified β-Zn_8Sb_7**	**β-Zn_8Sb_7**	**modified β-Zn_8Sb_7**	**β-Zn_8Sb_7**	**modified β-Zn_8Sb_7**
**Zn1**	0.1764(2)	0.264(4)	0.0274(4)	0.070(7)	1.1421(3)	1.139(3)
**Zn2**	0.0990(2)	0.111(3)	0.5133(5)	0.608(5)	0.2179(3)	0.243(4)
**Zn3**	0.1611(2)	0.097(3)	0.5163(5)	0.653(4)	1.0046(3)	1.022(3)
**Zn4**	0.1195(2)	0.143(3)	0.2534(5)	0.369 (6)	0.5172(3)	0.532(3)
**Zn5**	0.1093(2)	0.113(2)	0.7491(5)	0.693(4)	0.6262(3)	0.608(3)
**Zn6**	0.1007(3)	0.104(2)	0.0154(5)	0.015(4)	0.9265(3)	0.910(4)
**Zn7**	0.1584(3)	0.1584(3)	0.3626(7)	0.3626(7)	0.8128(4)	0.8128(4)
**Zn8**	0.1170(1)	0.1170(1)	0.445(2)	0.445(2)	0.7276(1)	0.7276(1)
**Zn9**	0.1573(3)	0.1573(3)	-0.1192(7)	-0.1192(7)	1.3311(4)	1.3311(4)
**Zn10**	0.1202(1)	0.1202(1)	-0.055(2)	-0.055(2)	0.4050(2)	0.4050(2)
**Sb1**	0	0	0.7268(3)	0.771(4)	0.3455(2)	0.324(3)
**Sb2**	0	0	0.5215(4)	0.5215(4)	0.5301(2)	0.5301(2)
**Sb3**	0	0	0.2159(3)	0.223(4)	0.7898(2)	0.799(3)
**Sb4**	0.2485(1)	0.237(1)	0.2194(3)	0.199(3)	0.9920(2)	1.001(2)
**Sb5**	0.3516(1)	0.3483(7)	0.2758(3)	0.281(2)	1.3320(1)	1.328(2)
**Sb6**	0.2416(1)	0.238(1)	-0.2885(3)	-0.289(2)	1.1503(2)	1.150(3)
**Sb7**	0.1669(1)	0.1649(7)	0.2386(3)	0.248(3)	1.3163(2)	1.321(2)
**Sb8**	0	0	0.0302(4)	0.061(3)	0.6011(2)	0.590(3)
**Sb9**	0	0	0.0317(3)	-0.011(3)	1.1036(2)	1.103(2)
**Sb10**	0	0	0.3914(3)	0.406(3)	1.0562(2)	1.057(2)

Table S6 Comparison of selective interatomic distances between the β-Zn_8Sb_7 phase and the modified β-Zn_8Sb_7 phase.

**Atom pairs**	**Distances (Å)**		**Atom pairs**	**Distances (Å)**	
	β-Zn_8Sb_7	modified β-Zn_8Sb_7		β-Zn_8Sb_7	modified β-Zn_8Sb_7
**Zn1 - Sb4**	2.653(4)	2.0661(2)	**Zn7 - Sb3**	2.653(5)	2.629(5)
**Zn1 - Sb5**	3.661(5)	3.1851(3)	**Zn7 - Sb4**	2.877(6)	2.985(6)
**Zn1 - Sb6**	2.633(4)	2.7137(4)	**Zn7 - Sb5**	2.811(6)	2.669(6)
**Zn1 - Sb7**	2.759(4)	3.1030(3)	**Zn7 - Sb6**	2.621(5)	2.688(5)
**Zn1 - Sb9**	2.697(3)	4.0896(3)	**Zn7 - Zn8**	1.402(2)	1.41(2)
**Zn2 - Sb1**	2.754(5)	2.3294(1)	**Zn8 - Sb2**	3.127(2)	3.17(2)
**Zn2 - Sb6**	2.771(4)	2.4049(1)	**Zn8 - Sb3**	2.616(2)	2.60(2)
**Zn2 - Sb7**	2.669(4)	2.9882(3)	**Zn8 - Sb5**	2.582(2)	2.48(2)
**Zn2 - Sb10**	2.711(4)	3.3019(3)	**Zn8 - Sb6**	2.635(2)	2.69(2)
**Zn3 - Sb4**	2.650(4)	4.0177(4)	**Zn9 - Sb1**	2.653(5)	2.530(5)
**Zn3 - Sb5**	2.730(4)	2.7024(3)	**Zn9 - Sb4**	2.609(5)	2.791(5)
**Zn3 - Sb6**	2.682(4)	2.7499(2)	**Zn9 - Sb6**	2.937(7)	2.940(6)
**Zn3 - Sb9**	4.636(5)	3.0914(3)	**Zn9 - Sb7**	2.776(6)	2.749(6)
**Zn3 - Sb10**	2.688(4)	2.4018(2)	**Zn9 - Zn10**	1.20(2)	1.21(2)
**Zn4 - Sb2**	2.747(4)	2.4608(1)	**Zn10 - Sb1**	2.584(2)	2.48(2)
**Zn4 - Sb6**	2.704(4)	2.4348(1)	**Zn10 - Sb4**	2.595(2)	2.72(2)
**Zn4 - Sb7**	2.660(4)	2.9040(3)	**Zn10 - Sb7**	2.632(2)	2.61(2)
**Zn4 - Sb8**	2.710(4)	3.2570(3)	**Zn10 - Sb8**	3.15(2)	3.14(2)
**Zn5 - Sb2**	2.700(4)	2.3733(1)	**Sb1 - Sb2**	2.839(4)	3.2559(3)
**Zn5 - Sb4**	2.748(4)	2.7801(2)	**Sb3 - Sb8**	2.801(4)	2.9698(3)
**Zn5 - Sb5**	2.695(4)	2.9133(3)	**Sb5 - Sb7**	2.798(2)	2.8011(2)
**Zn5 - Sb8**	2.743(4)	3.2507(3)	**Sb9 - Sb10**	2.845(4)	3.1655(4)
**Zn6 - Sb3**	2.780(5)	2.6468(2)			
**Zn6 - Sb4**	2.850(4)	2.7223(1)			
**Zn6 - Sb5**	2.652(5)	2.5591(3)			
**Zn6 - Sb9**	2.720(4)	2.9593(3)			

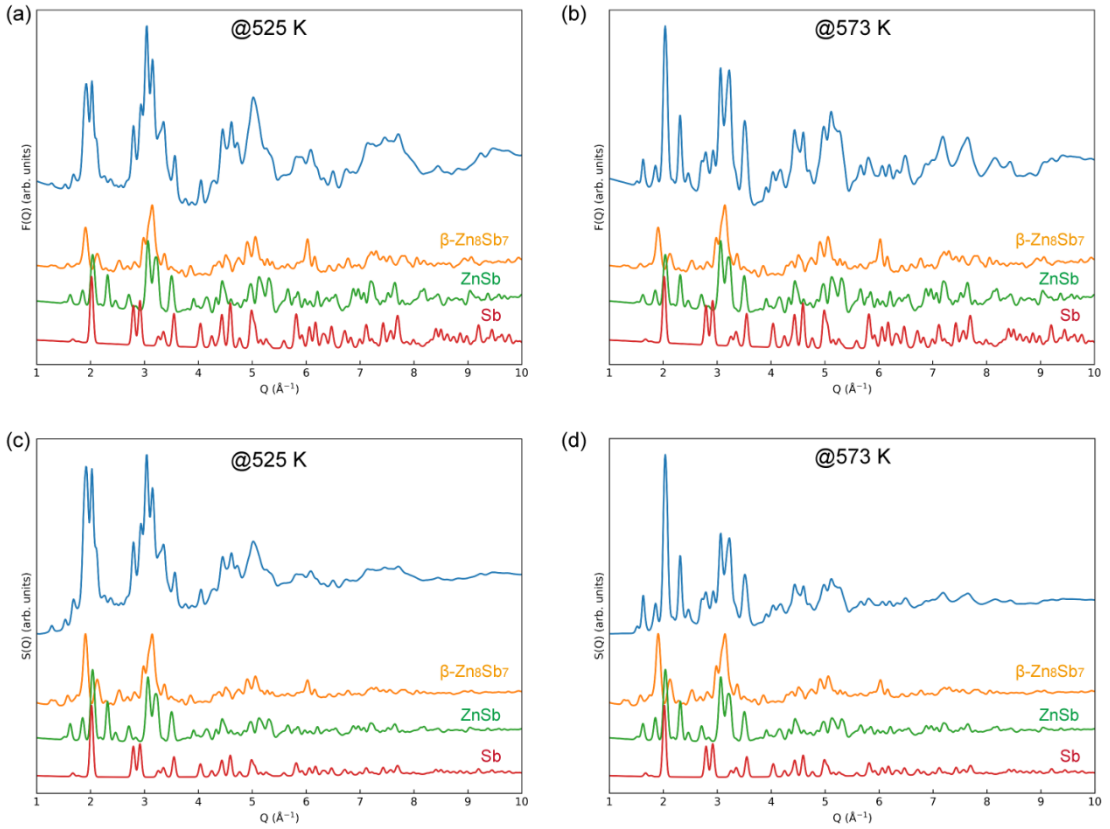

Figure S8 (a-b) $F(Q)$ and (c-d) $S(Q)$ for the ZSA2 film in comparison with the calculated patterns of β-Zn_8Sb_7 phase (space group $Pmn2_1$), ZnSb phase (space group $Pbca$) and Sb (space group $R\overline{3}c$).

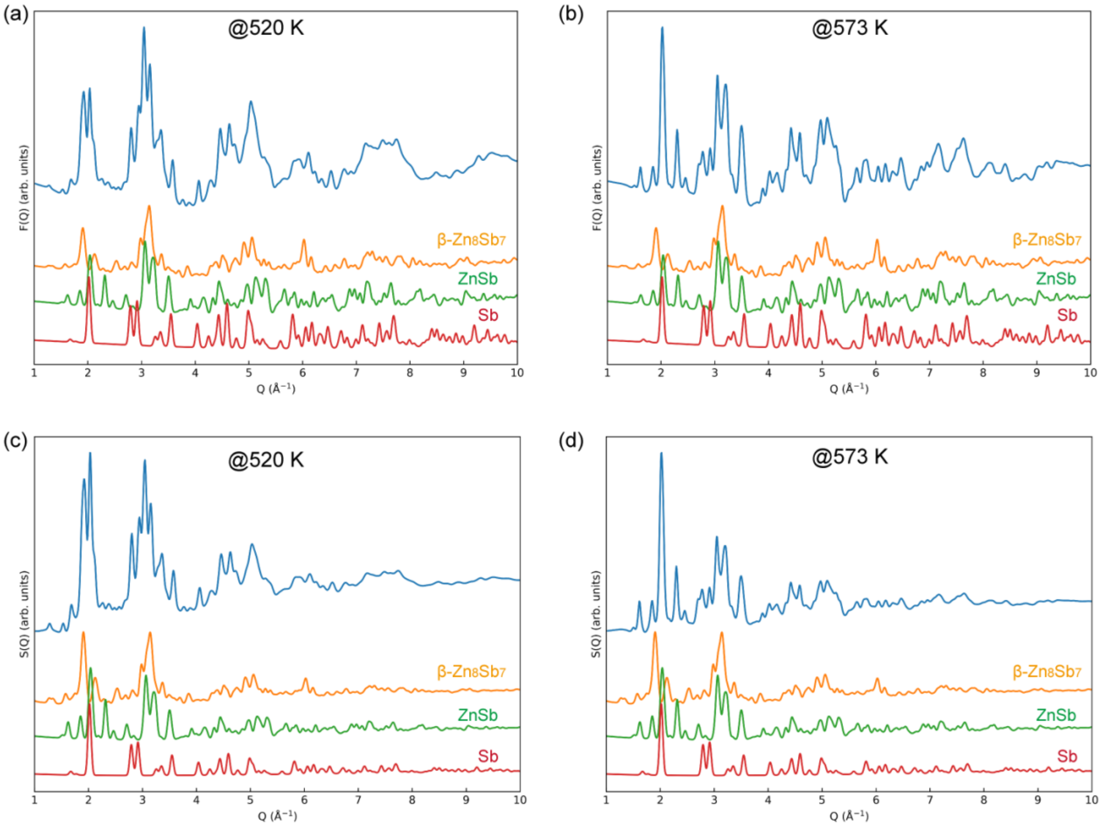

Figure S9 (a-b) $F(Q)$ and (c-d) $S(Q)$ for the ZSA3 film in comparison with the calculated patterns of β-Zn_8Sb_7 phase (space group $Pmn2_1$), ZnSb phase (space group $Pbca$) and Sb (space group $R\bar{3}c$).

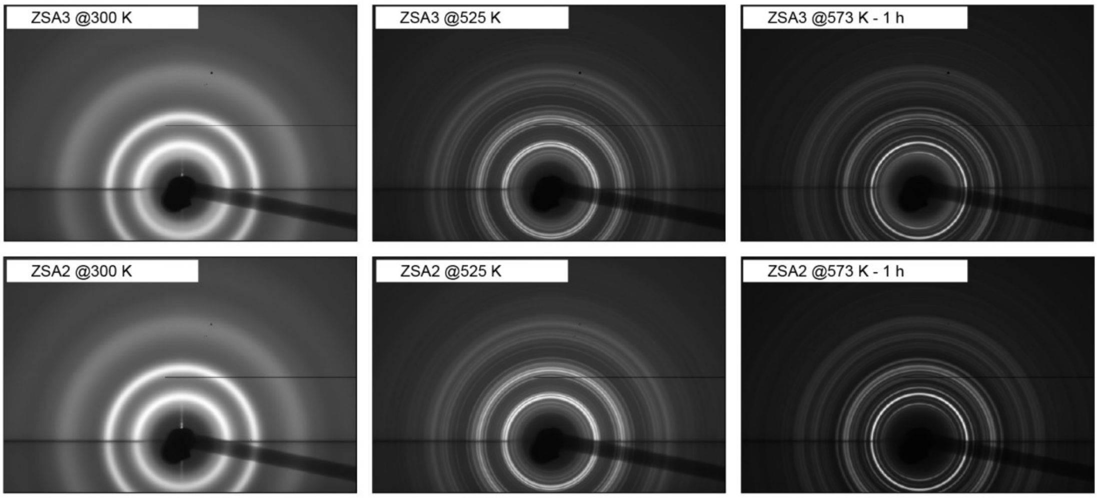

Figure S10 Zoom-in of the un-masked, raw grazing incidence diffraction images of the amorphous phase (@300 K), intermediate phase (@525 K), and ZnSb phase (after 1 hour's annealing @573 K) for the two thin films of ZSA2 and ZSA3.

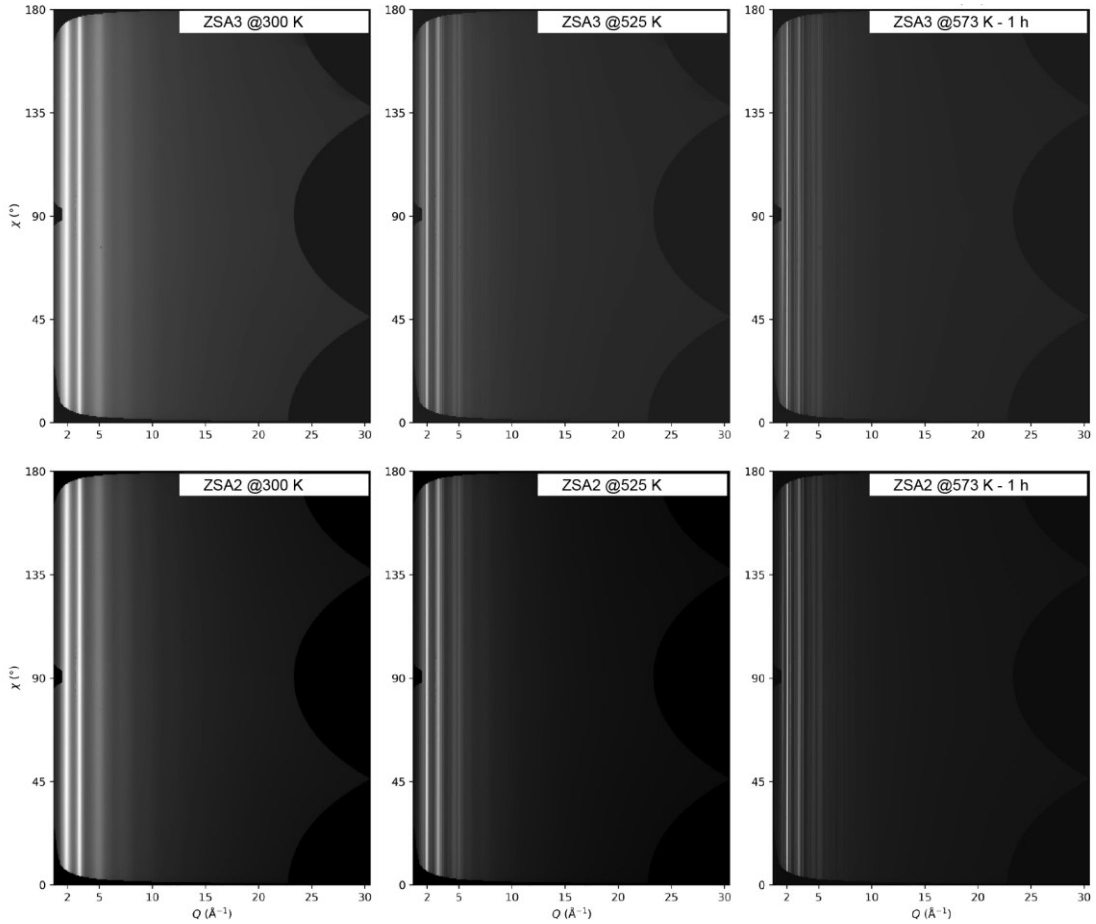

Figure S11 Unwrapped 2D diffraction pattern, *i.e.* unwrapped intensity plotted as a function of Q and the azimuth for the two thin films of ZSA2 and ZSA3. Black regions are masked out due to lack of detector coverage and pixel errors on the detector. The intensities at each position in Q are spread evenly over the whole azimuth meaning there is no pronounced preferred orientation.

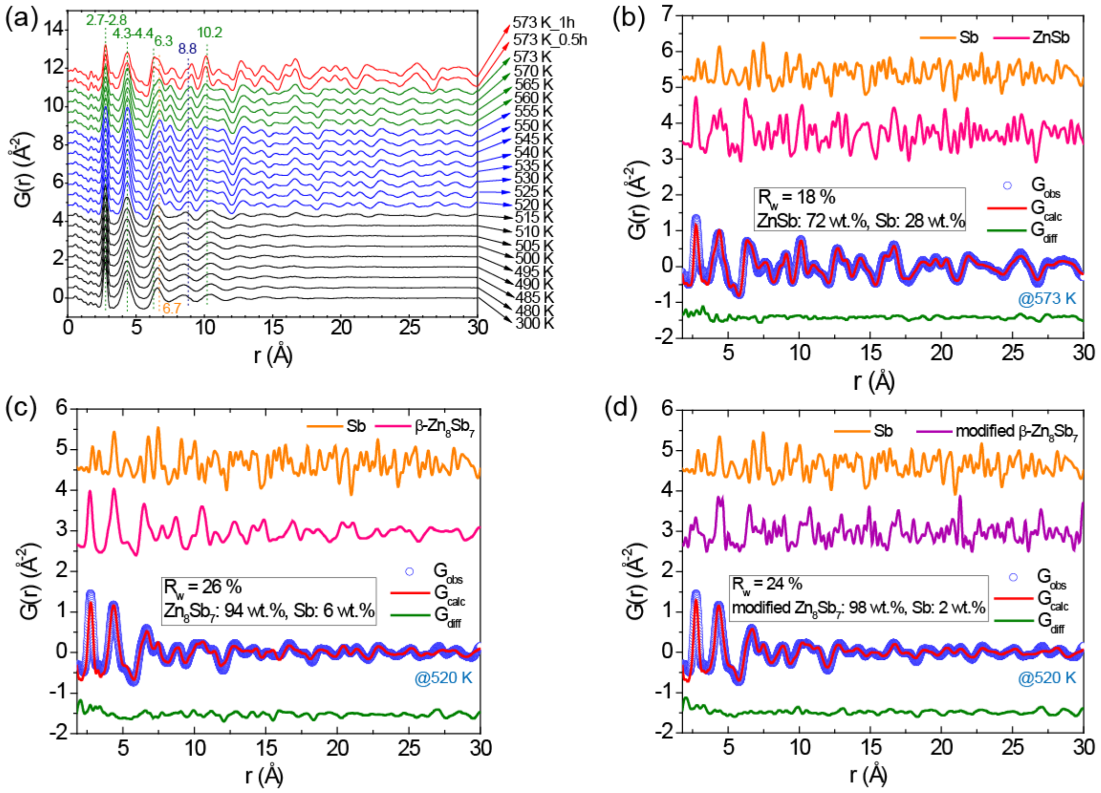

Figure S12 (a) *In situ* GI-PDFs from total scattering at some selected measurement temperatures for the ZSA3 film over the range of 0-30 Å. The corresponding PDF data over 0-15 Å and 0-60 Å are shown in Figure S13c-d. Fits (red solid curve) to the experimental PDF data (blue open circles) at 573 K after a dwell time of 1 h (b) and at 520 K (c and d). In (b), (c) and (d), the difference between the experimental data and model fit is shown in green, and the refined parameters for the fits are given in Table S7, S8 and S9.

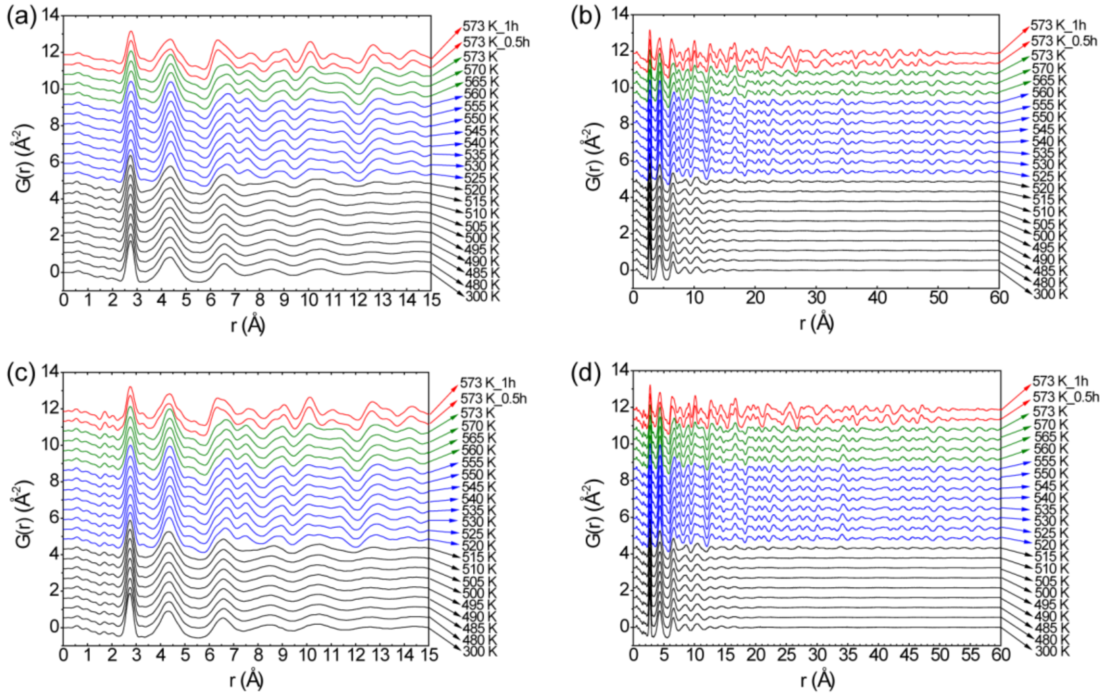

Figure S13 (a) *In situ* GI-PDFs from total scattering at some selected measurement temperatures for the (a-b) ZSA2 and (c-d) ZSA3 films shown in the *r* range of 0-15 Å and 0-60 Å.

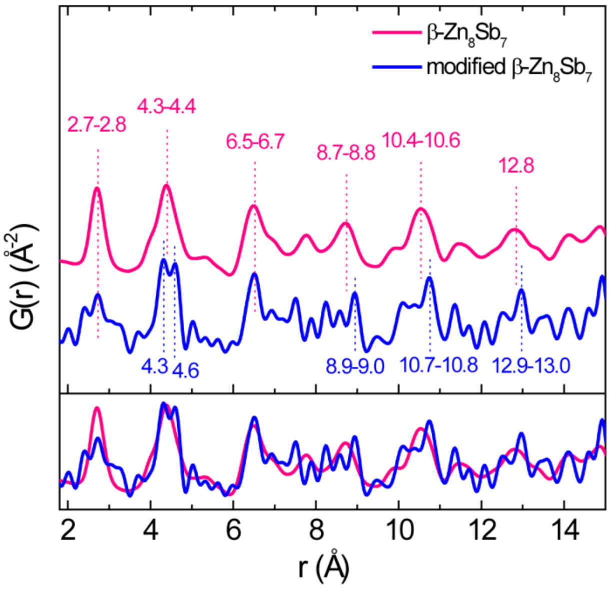

Figure S14 Comparison of the theoretical PDF patterns of the β-Zn_8Sb_7 phase and the modified β-Zn_8Sb_7 phase.

Table S7 Parameters of the PDF fitting using the model with two phases (the ZnSb phase and Sb phase) for the film samples. Estimated error was not given for the parameter with a fixed value.

Film sample	*T* (K)	Model	R_w (%)	ZnSb phase						
				a (Å)	*b* (Å)	*c* (Å)	Weight fraction	$U_{iso,Zn}$ (Å^2)	$U_{iso,Sb}$ (Å^2)	δ_2 (Å^2)
ZSA2	573	ZnSb + Sb	18.2	6.21(2)	7.73(4)	8.07(4)	77(5)%	0.026(18)	0.015(4)	5(2)
ZSA3	573	ZnSb + Sb	17.7	6.24(2)	7.76(3)	8.12(4)	72(5)%	0.025(17)	0.013(4)	5(2)
ZSA1_423K	573	ZnSb + Sb	9.8	6.26(1)	7.81(2)	8.15(3)	96(5)%	0.016(8)	0.009(2)	5(2)
ZS2	568	ZnSb + Sb	20.9	6.25(3)	7.78(4)	8.11(4)	67(8)%	0.055(43)	0.026(9)	5(2)

**Sb phase**					
***a* (Å)**	***c* (Å)**	**Weight fraction**	$U_{11,Sb} = U_{22,Sb}$ **(Å^2)**	$U_{33,Sb}$ **(Å^2)**	δ_2 **(Å^2)**
4.29(2)	11.3(2)	23(5)%	0.020	0.034	7(3)
4.31(2)	11.4(1)	28(5)%	0.020	0.034	6(4)
4.33(2)	11.5(8)	4(5)%	0.020	0.034	7
4.32(1)	11.4(1)	33(8)%	0.027(7)	0.047(26)	7(1)

Table S8 Parameters of the PDF fitting using the model with two phases (the β-Zn_8Sb_7 phase and Sb phase) for the ZSA2 and ZSA3 films. Estimated error was not given for the parameter with a fixed value.

Film sample	*T* (K)	Model	R_w (%)	Zn_8Sb_7 phase						
				a (Å)	*b* (Å)	*c* (Å)	Weight fraction	$U_{iso,Zn}$ (Å^2)	$U_{iso,Sb}$ (Å^2)	δ_2 (Å^2)
ZSA2	525	Zn_8Sb_7 + Sb	24.3	14.77(13)	7.54(8)	13.59(13)	93(4)%	0.022	0.014	5
ZSA3	520	Zn_8Sb_7 + Sb	25.6	14.83(15)	7.51(8)	13.57(11)	94(4)%	0.022	0.014	5

**Sb phase**					
***a* (Å)**	***c* (Å)**	**Weight fraction**	$U_{11,Sb} = U_{22,Sb}$ **(Å^2)**	$U_{33,Sb}$ **(Å^2)**	δ_2 **(Å^2)**
4.30(4)	11.1(3)	7(4)%	0.008	0.019	8
4.29(6)	11.2(5)	6(4)%	0.008	0.019	8

Table S9 Parameters of the PDF fitting using the model with two phases (the modified β-Zn_8Sb_7 phase and Sb phase) for the ZSA2 and ZSA3 films. Estimated error was not given for the parameter with a fixed value.

Film sample	T (K)	Model	R_w (%)	Zn_8Sb_7 phase						
				a (Å)	b (Å)	c (Å)	Weight fraction	$U_{iso,Zn}$ (Å^2)	$U_{iso,Sb}$ (Å^2)	δ_2 (Å^2)
ZSA2	525	modified Zn_8Sb_7 + Sb	25.2	14.84(14)	7.58(10)	13.43(16)	97(5)%	0.019	0.018	5
ZSA3	520	modified Zn_8Sb_7 + Sb	23.6	14.80(11)	7.58(7)	13.57(14)	98(4)%	0.019	0.018	5

**Sb phase**					
a **(Å)**	***c*** **(Å)**	**Weight fraction**	$U_{11,Sb} = U_{22,Sb}$ (Å^2)	$U_{33,Sb}$ (Å^2)	δ_2 (Å^2)
4.35(11)	11.0(9)	3(5)%	0.008	0.019	8
4.32(13)	11.1(8)	2(4)%	0.008	0.019	8

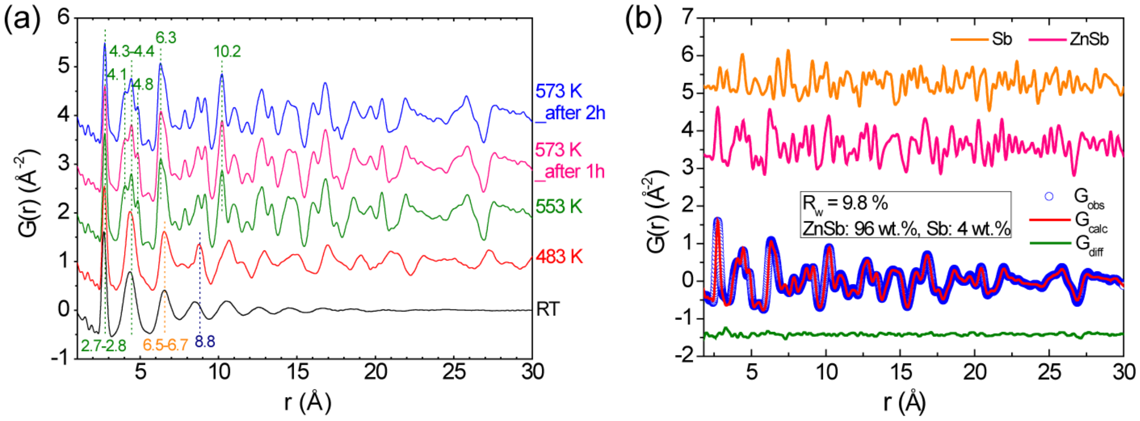

Figure S15(a) *Ex situ* GI-PDFs from total scattering at different annealing temperatures for the ZSA1_423K film over the range of 1-30 Å. (b) Fit (red solid curve) to the experimental PDF data (blue open circles) at 573 K after a dwell time of 1 h. In (b), the difference between the experimental data and model fit is shown in green, and the refined parameters for the fits are given in Table S7.

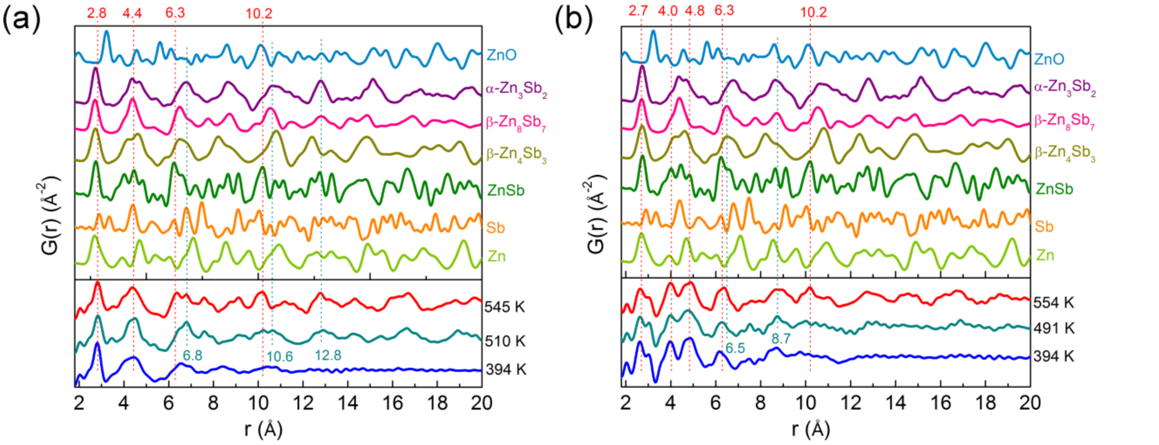

Figure S16 (a) and (b) show PDF patterns (1.8-20 Å) at selected surface temperatures for the ZS2 and ZS3_373K films, respectively. The corresponding PDF patterns over 0-15 Å and 0-70 Å are shown in Figure S17. The theoretical PDFs of the reported phases are plotted for comparison.

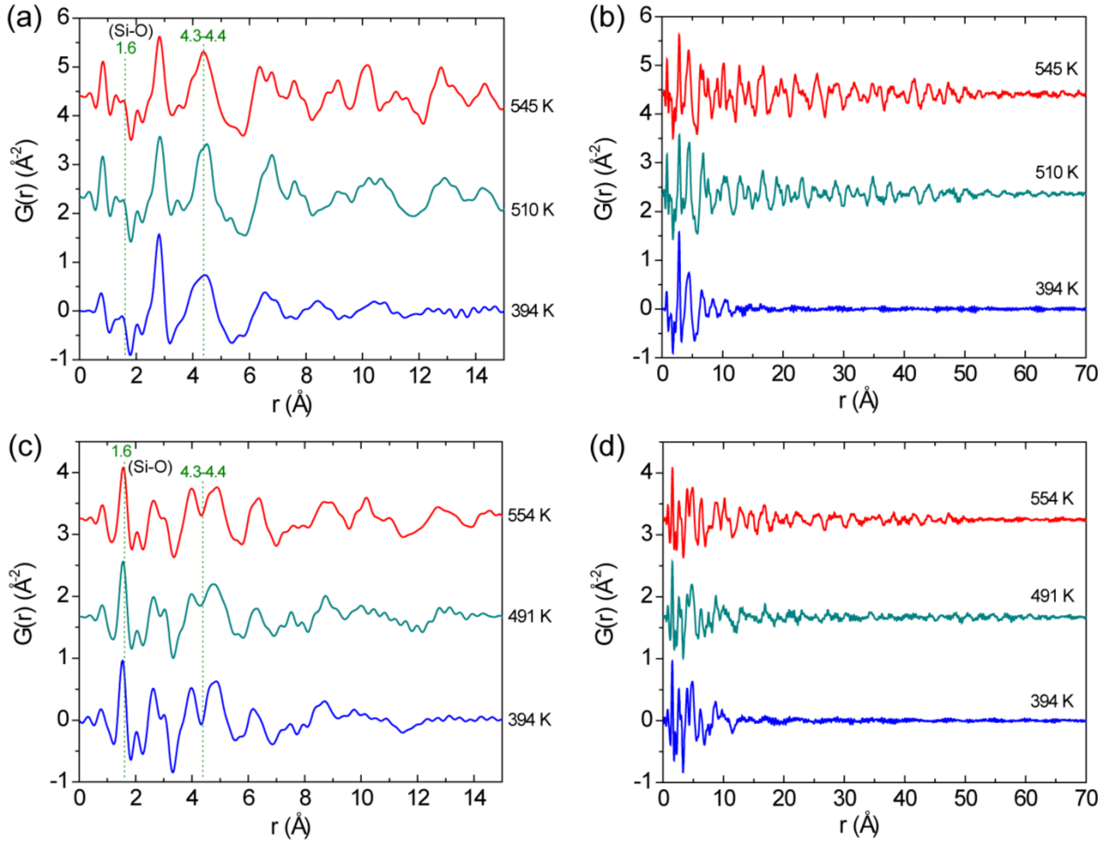

Figure S17 PDF patterns at selected surface temperatures for the (a-b) ZS2 and (c-d) ZS3_373K films shown in the *r* range of 0-15 Å and 0-70 Å.

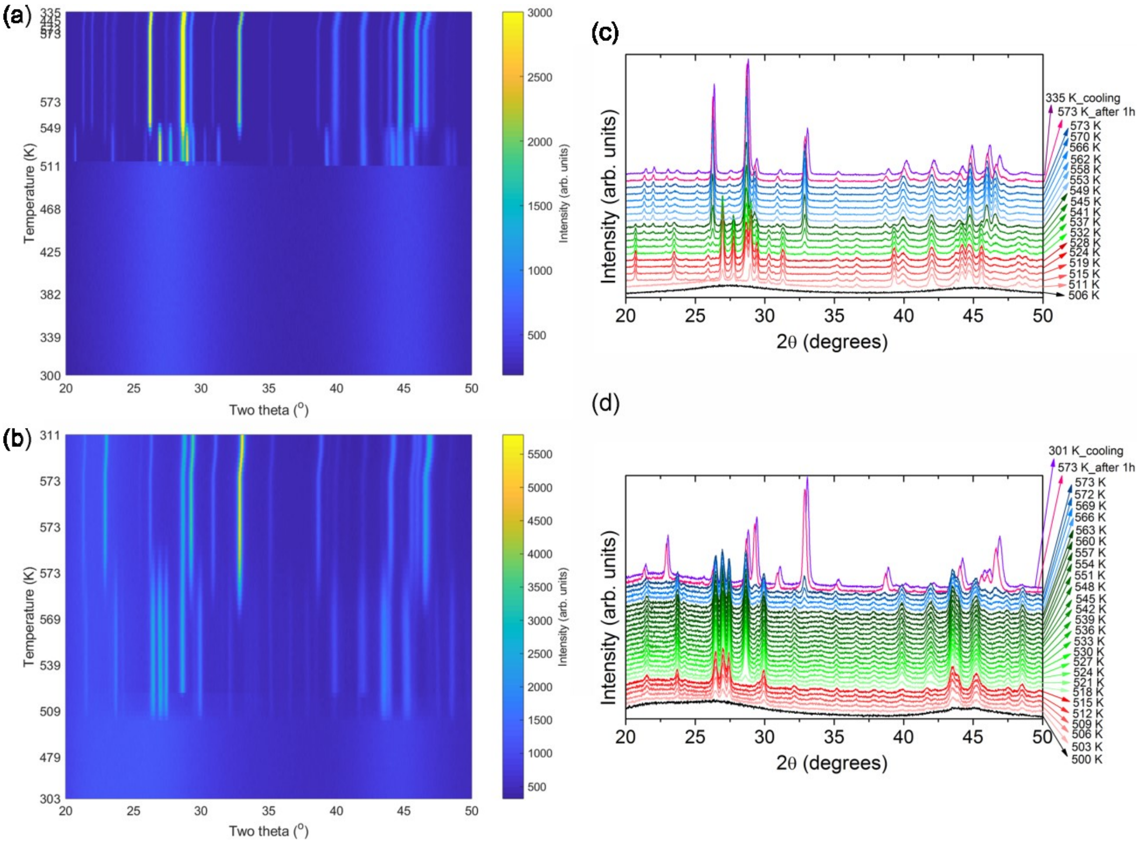

Figure S18 Contour plots of in-house *in situ* XRD data (Cu-Kα source) for the two undoped film samples: (a) ZS2 (using the Ud#2 target) and (b) ZS3_RT (using the Ud#3 target). (c) and (d) show the corresponding XRD patterns at selected temperatures for the two films of ZS2 and ZS3_RT, respectively.

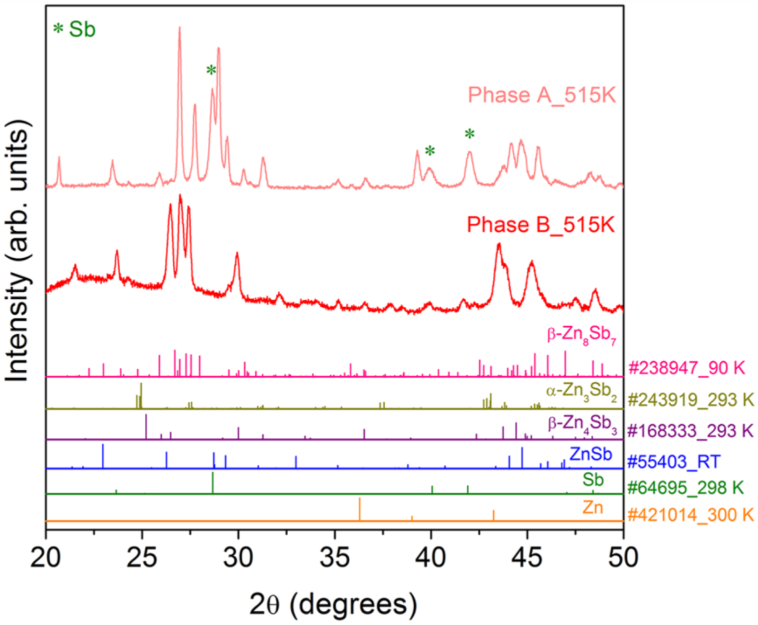

Figure S19 XRD patterns of the two metastable phases (A and B) appearing in the corresponding films during annealing.

Notes for Figures S18 and S19: A clear structural change of the two as-deposited undoped films can be seen from Figure S18a-b during the heat treatment. Both ZS2 and ZS3_RT are amorphous films deposited at RT, but they start to crystallize when the annealing temperatures reach certain values. Figure S18a and s18c shows that for the ZS2 film with a starting Sb composition of 60 at.%, the formation of a metastable phase A accompanied with an Sb phase occurs at 511 K, and phase A disappears at 553 K. At around 532 K, ZnSb forms at the expense of phase A and co-exists with the Sb phase after being held at 573 K for 1 h and cooled down to RT. Figure S18b and S18d shows that the ZS3_RT film with a starting Sb composition of 49 at.% crystallizes into an intermediate phase B at 503 K, and the Sb phase subsequently emerges. When the temperature reaches above 566 K, the ZnSb phase starts to form at the expense of phase B. After being kept at 573 K for 1 h, the metastable phase B has completely transformed, and the ZnSb phase and a small amount of the Sb phase remain in the ZS3_RT film after being cooled down to RT. The critical temperatures for different phases in the films of ZS2 and ZS3_RT are tabulated in Table S10. Interestingly, it is found that the formation of the two metastable phases (A and B) is always followed by the emergence of an Sb phase at some stage. In Figure S19, it is observed that the amount of the Sb phase relative to the metastable phase A is impressively higher than that relative to the metastable phase B.

Table S10 The temperatures at which the metastable phase B, Sb phase or ZnSb phase appears and disappears in the two thin films (ZS2 and ZS3_RT) grown by using different sputtering targets.

Film sample		ZS2 (s1)	ZS3_RT
**Target**		ZnSb (Ud#2)	Zn_4Sb_3 (Ud#3)
**Film composition**		Zn:Sb = 40:60 = 0.67	Zn:Sb = 51:49 = 1.04
**T_{max} (K)**		573	573
**phase A**	**T_{appear} (K)**	511	-
	**$T_{disappear}$ (K)**	553	-
**phase B**	**T_{appear} (K)**	-	503
	**$T_{disappear}$ (K)**	-	573
**Sb**	**T_{appear} (K)**	511	503
**ZnSb**	**T_{appear} (K)**	532	566

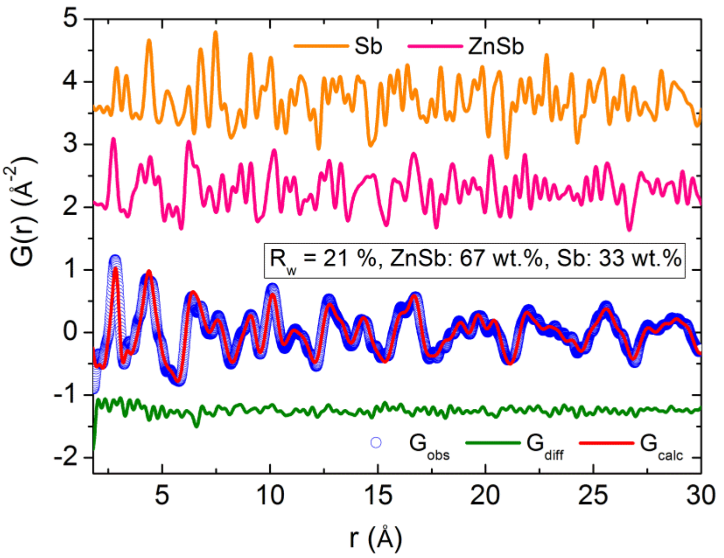

Figure S20 PDF fitting (red curve) to the experimental data (blue open circles) for the ZS2 film at 568 K. The green line shows the difference curve. For comparison, the theoretical PDFs of the ZnSb and Sb phases are also plotted (pink and orange curves, respectively). The details for the refined parameters can be seen in Table S7.
